# Chrono-nutrition and sleep: lessons from the temporal feature of eating patterns in human studies - A systematic scoping review

**DOI:** 10.1016/j.smrv.2024.101953

**Published:** 2024-05-18

**Authors:** Oussama Saidi, Emmanuelle Rochette, Lou Dambel, Marie-Pierre St-Onge, Pascale Duché

**Affiliations:** a JAP2S Laboratory, Toulon University, F-83041, Toulon, France; b Department of Pediatrics, Clermont-Ferrand University Hospital, F-63000, Clermont-Ferrand, France; c Clermont Auvergne University, INSERM, CIC 1405, CRECHE Unit, F-63000, Clermont-Ferrand, France; d Center of Excellence for Sleep & Circadian Research and Division of General Medicine, Department of Medicine, Columbia University Irving Medical Center, New York, NY, USA, 10032

**Keywords:** Meal timing, Breakfast skipping, Late eating, Meal delay, Ramadan, Intermittent fasting, Time-restricted eating, Irregular meal pattern, Meal frequency, Sleep health

## Abstract

An emerging field of research has revealed a bidirectional relationship between sleep and diet, highlighting the potential role of a healthy diet in improving sleep. However, the impact of chrono-nutrition on sleep remains less explored. Here we conducted a systematic scoping review, considering the multiple dimensions of chrono-nutrition, to describe the extent, range, and nature of the existing literature in this area (PROSPERO: CRD42021274637). There has been a significant increase in the literature exploring this topic over the past six years (almost 67 % of the evolving literature). A breakdown of the included studies was performed according to three major chrono-nutritional dimensions: meal timing [n = 35], irregular eating patterns [n = 84], and frequency of eating occasions [n = 3]. Meal timing included three sub-dimensions: breakfast skipping [n = 13], late eating [n = 16], and earlier vs later meals schedules [n = 6]. Irregular meal patterns included three subdimensions: diurnal fasting [n = 65], intermittent fasting [n = 16], and daily meal patterns [n = 3]. Frequency was the least studied dimension (n = 3). We provided a synthetic and illustrative framework underlining important preliminary evidence linking the temporal characteristics of eating patterns to various facets of sleep health. Nonetheless, much work remains to be done to provide chrono-nutrition guidelines to improve sleep health in the general population.

## 1. Introduction

Over the past two decades, a growing body of evidence has revealed a complex, bidirectional relationship between sleep and diet. Numerous studies have demonstrated that sleep deprivation can significantly affect food intake and contribute to dietary overconsumption (an approximate 25 % increase in total energy intake) through multiple pathways. For example, sleep deprivation has been shown to disrupt appetite hormones and increase the activation of brain regions associated with reward in response to food stimuli [1,2]. On the other hand, diet has been shown to significantly influence sleep physiology [3,4]. Consumption of an unhealthy diet high in saturated fat and sugar has been associated with altered sleep quality, disruption of sleep oscillatory patterns, and reduction of slow-wave activity [5,6]. Conversely, various dietary strategies, including consumption of foods rich in melatonin, serotonin, and tryptophan, as well as adjustments in macronutrient proportions or dietary glycemic index, have shown the potential to significantly improve sleep [7–9].

More recently, in addition to considering the quantity and quality of food, the temporal characteristics of eating habits have garnered attention from researchers, under the umbrella term “chrono-nutrition” [10]. This approach advocates aligning eating patterns with the body’s natural circadian rhythms [11]. The circadian system orchestrates digestion and absorption by regulating the secretion and activity of several gut hormones and enzymes, as well as gastrointestinal motility, which follow a diurnal motor rhythm in contrast to nocturnal rest [12]. The concept of chrono-nutrition relies on a circadian system that drives wakefulness and eating during the biological day while promoting sleep and fasting during the biological night [13]. However, eating patterns are influenced by a variety of factors, including food availability, hunger/satiety, social habits, and convenience [11]. Urbanization has introduced drastic changes in the temporal characteristics of eating patterns over the last century. Skipping breakfast, delaying meals, and eating late at night have all been described as growing challenges of modern society [14]. Considering that meal intake serves as a circadian time cue [15], it has been suggested that mistimed meals could disrupt the endogenous clock, resulting in circadian desynchronization and metabolic disruption [13]. Some evidence directly links the timing of certain eating patterns to obesity [16]. For instance, an examination of time-of-day energy intake and its association with obesity was conducted in a previous review, including eight cross-sectional studies (three involving children) and two longitudinal cohort studies (one involving children) [17]. The collective findings support the association between higher energy intake in the evening and the development of obesity.

To date, most research efforts have concentrated on investigating the effects of chrono-nutrition on metabolism. However, given the growing awareness of the bidirectional relationship between diet and sleep and the critical role of sleep for health [18], it is possible that sleep may serve as a mediator between the temporal characteristics of eating patterns and the development of obesity and cardiometabolic diseases [19]. Consequently, sleep may be the missing link, and chrono-nutritional regimens could potentially enhance metabolic regulation by improving sleep health. Due to its practicality and ease of implementation, chrono-nutrition holds promise as a viable strategy to enhance sleep quality in the general population. However, the current literature remains scattered, lacking consensus on the potential changes in sleep from exposure to a specific eating schedule. Therefore, this review aims to provide a comprehensive overview and synthesis of the evolving evidence regarding the effects of the temporal characteristics of eating patterns on sleep. Through a meticulous examination of the current state of the art, we identified gaps and outlined potential directions for future research in this area.

## 2. Methods

### 2.1. Search strategy

We defined chrono-nutrition based on three major dimensions, as used in scientific literature: timing (actual time of day), regularity (consistency of eating routine throughout the day or from day-to-day), and frequency (number of meals per day) of food intake [14,20]. Given the breadth of the topic, we performed a systematic scoping review method to assess the effect of chrono-nutrition on sleep. The recommendations of the PRISMA statement for scoping reviews (Preferred Reporting Items for Systematic Reviews and Meta-Analyses; www.prismastatement.org/Extensions/ScopingReviews) were followed [21]. The protocol is registered on PROSPERO (CRD42021274637).

The search strategy identified peer-reviewed articles published in English between 1998 to December 2022 in PubMed (MEDLINE), ScienceDirect (Elsevier) and Scopus. A search algorithm was formulated based on subject headings and medical subject headings for each chrono-nutrition dimension and sleep health outcomes. Only the combination of each chrono-nutrition dimension with sleep-related terms was considered relevant (Table S1).

The search strategy was developed according to the PICO framework (Population, Intervention/Exposure, Comparison (if any) and Outcome) [22] where the population [P] was human subjects aged >2 years, the intervention/exposure [I] was any time features in eating patterns (meal timing, irregular meal pattern, and frequency), and the outcome [O] included sleep health-related measures. There were no restrictions on study design, quality, and location. We excluded [1]: non-primary research papers (e.g., reviews, case reports, editorials, and conference papers) [2], studies conducted in animal and/or cell models [3], studies conducted in shift workers or populations with eating disorders (e.g., night eating syndrome), and [4] studies only reporting sleep respiratory events.

### 2.2. Screening

Research Information System formatted references were exported from the databases, and imported into the CADIMA software [23] where a 2-stage screening process was used to select the studies to be included after duplicate removal. Two authors (OS and LD) performed the first screening based on the title and abstract. Screening agreement was achieved (Cohen’s Kappa >80 %) for the first 10 % of references. Then, a full text screening of selected articles was undertaken. Conflictual classification of a study was resolved by discussion. In cases of missing data, the corresponding author of the published study was contacted. If no response was obtained within one month, the study was excluded.

### 2.3. Data extraction

Included studies were summarized independently by the first three authors (OS, ER, and LD). Data extracted were then verified by two authors to ensure consistency in the process. A standardized eligibility criteria sheet including information regarding (1) first authors and year of publication, (2) design, (3) population/sample size and characteristics, (4) duration, (5) sleep outcomes (self-reported and/or objective), (6) main findings was developed (Tables S2, S3, and S4). Questionnaires and diaries were coded as self-reported measures while accelerometer, electroencephalography and polysomnography (PSG) data were coded as objective.

### 2.4. Data analysis and synthesis

Keeping in mind the exploratory nature of this review, a breakdown of the included studies according to chrono-nutrition dimensions and sub-dimension was performed in agreement with current recommendations [14,20]. Sub-dimension for timing were breakfast skipping, late night eating, and earlier vs later meals schedules. Studies of irregular meal patterns were classified into three major sub-dimensions: diurnal fasting, intermittent fasting, and day-to-day meal patterns. No sub-dimensions were proposed for meal frequency. The full list of references for the included studies is available in the Supporting file (online supplement).

A metadata approach was developed to draw a structured mapping of evidence. Performing a meta-analysis was not applicable given the dissimilarity of included studies (e.g., design, data analysis, sample characteristics) that could not be quantitively combined. Therefore, a qualitative and descriptive approach was followed to synthesize the available evidence for each sub-dimension of chrono-nutrition. To better synthesize the level of evidence, a coding scheme allowing classification of the level of agreement between the existing studies using the traffic light approach was elaborated. A detailed tabular representations of each study sleep outcomes are also available for meal timing dimension (Fig. S1), diurnal fasting, and intermittent fasting sub-dimensions (Fig. S2).

## 3. Results

### 3.1. Overview of the included studies

**Fig. 1.**
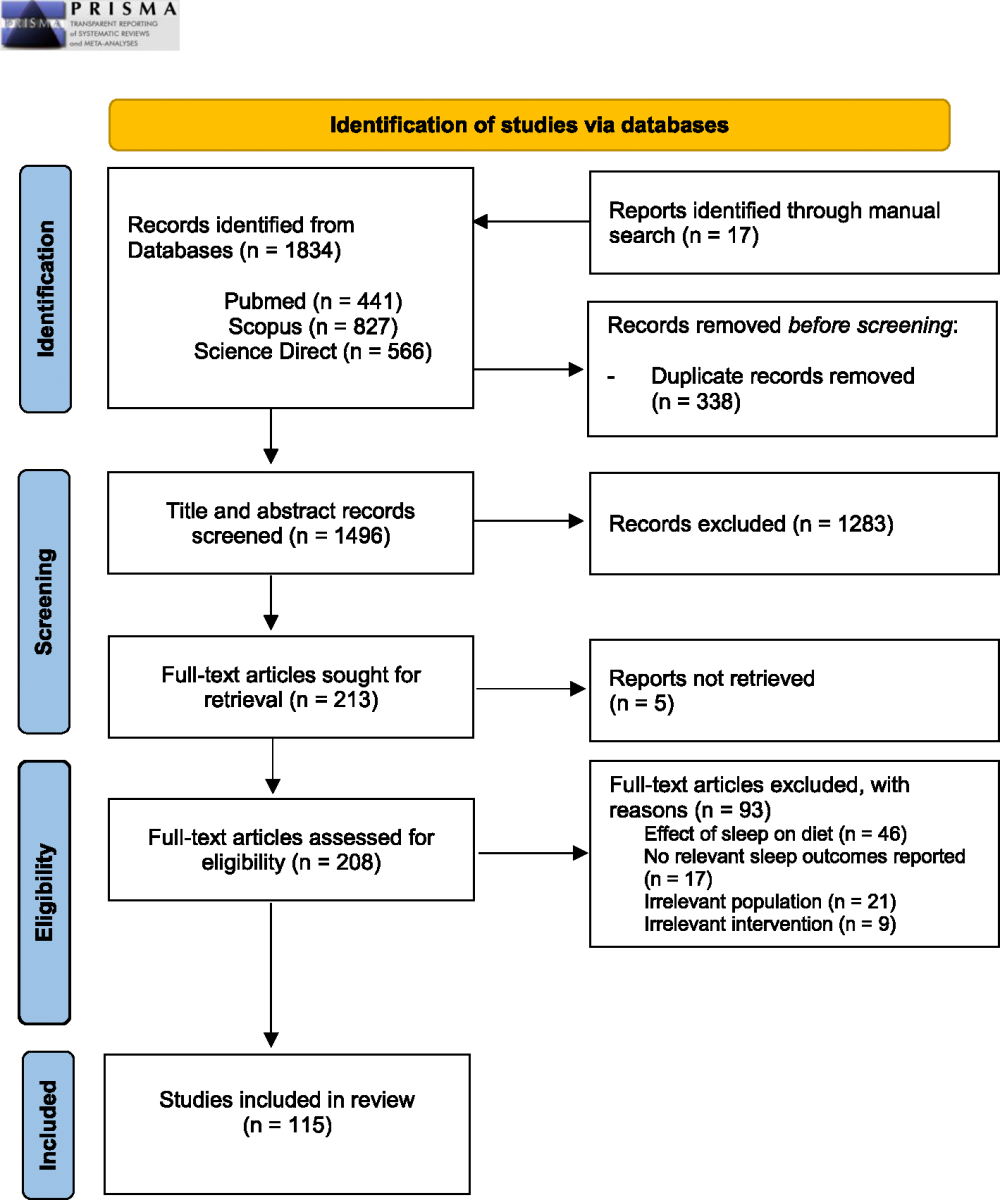
PRISMA Flow chart of study literature search. *From:* Page MJ, McKenzie JE, Bossuyt PM, Boutron I, Hoffmann TC, Mulrow CD et al. The PRISMA 2020 statement: an updated guideline for reporting systematic reviews. BMJ 2021; 372:n71. doi: 10.1136/bmj.n71.

A total of 1834 records were identified through initial electronic database search along with manual search (Fig. S3). Upon removal of duplicates, 1496 publications entered title/abstract level screening; 208 studies underwent full-text assessment and almost half (49.5 %) were not considered because they assessed the effect of sleep on diet (inverse relationship). Seventeen studies (18.3 %) were excluded because they focused on non-relevant circadian or sleep outcomes, 21 studies (22.6 %) because they involved populations with eating disorders, mostly night eating syndrome, and 9 studies (9.6 %) because of irrelevant intervention. A final sample of 115 publications was analyzed (see Fig. 1).

**Fig. 2.**
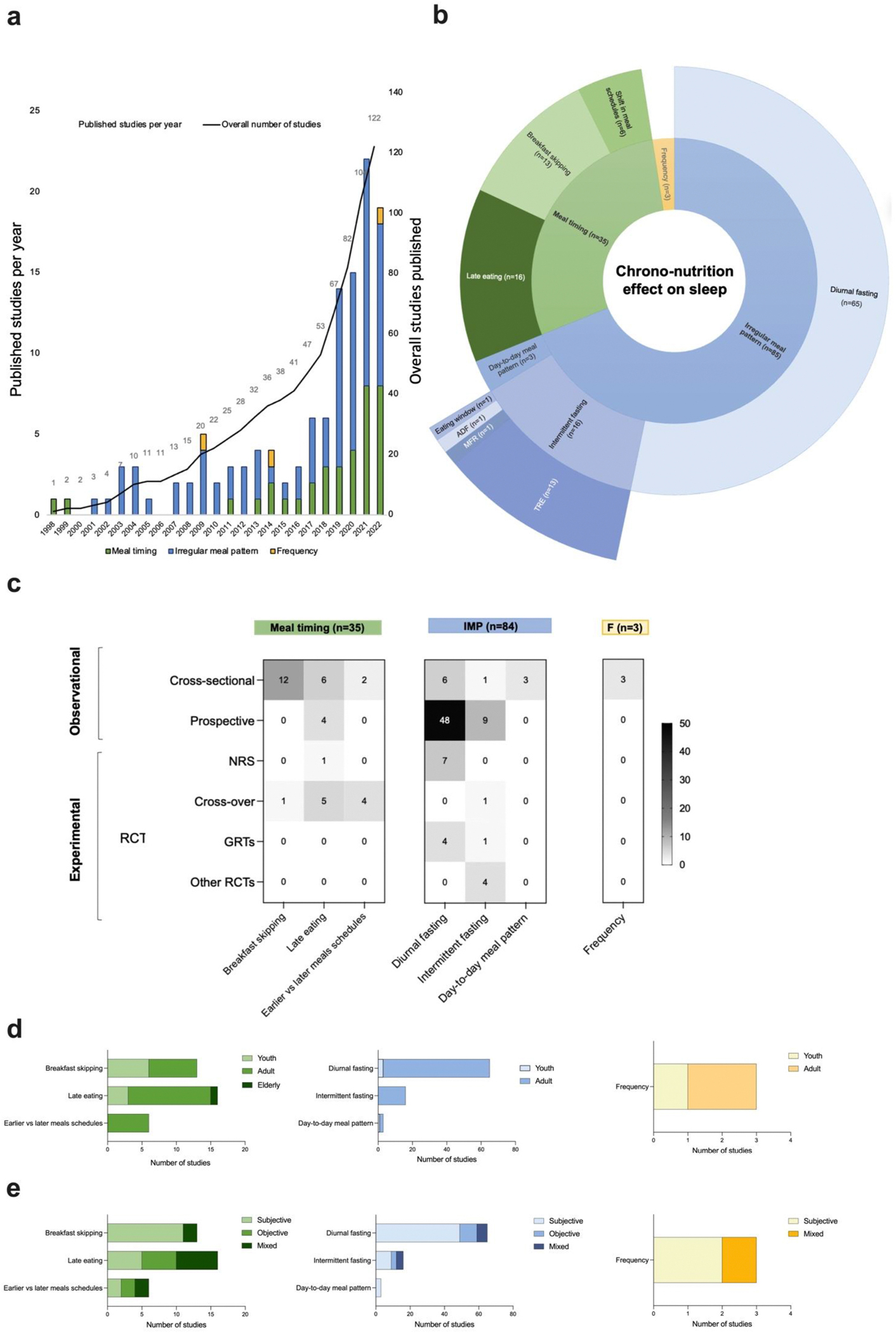
Meta-data from the review process. (**a**) Breakdown of included studies according to chrono-nutrition dimensions and sub-dimensions. (**b**) Original research papers published over time. (**c**) Heatmap of the included studies according to design broken down into chrono-nutrition dimensions. (**d**) Participants age groups in the included studies according to chrono-nutrition sub-dimensions. (**e**) Sleep assessment methods in the included studies according to chrono-nutrition sub-dimensions. * NB: Three studies were included in three sub-dimensions and one study was included in two subdimensions, increasing the overall number of studies per chrono-nutrition dimension to 123 studies. ADF: Alternated day fasting, F: frequency GRTs: parallel group-randomized trials, IMP: irregular meal pattern, MFR: Modified-fasting regimens, NRS: non randomized studies, RCTs: randomized controlled trials, TRE: time-restricted eating.

Fig. 2 provides meta-data of the included studies according to chrono-nutrition dimensions. Three studies were relevant to three subdimensions and one study was relevant for two sub-dimensions, increasing the overall number of items per chrono-nutrition dimensions to 122. A total of 35 studies (28.7 %) related to meal timing (n = 13 breakfast skipping, n = 16 late eating, and n = 6 shift in meal schedules, Table S2), 84 (68.8 %) related to irregular meal pattern (n = 65 diurnal fasting, n = 16 intermittent fasting, Table S3), and 3 (2.5 %) related to meal frequency (Table S4).

Of the 122 categorized studies, 94 (77 %) were observational while 29 (23 %) were interventional (Fig. 2c). Of the randomized controlled trials (RCTs), 10 investigated the effect of meal timing, 10 studied the effect of irregular meal patterns, and none tested the effect of meal frequency on sleep. Youth were less represented than adult populations especially in relation to irregular meal pattern dimensions where only 3 (3.5 %) of 85 studies included adolescents (Fig. 2d). Most of the included studies (66.3 %, n = 81) relied on a self-reported assessment of sleep.

### 3.2. Meal timing

#### 3.2.1. Breakfast skipping

The effect of skipping breakfast on sleep was investigated in 13 studies (Fig. S1a). Most studies used self-report methods, two used both self-report and objective methods. Half of the studies were carried out in youth. All studies used an observational approach except for one.

Most cross-sectional studies of children and adolescents consistently report delayed bedtimes, shorter sleep duration, and lower self-reported sleep quality in breakfast skippers relative to breakfast eaters. Studies in adults also report delayed bedtimes and poorer sleep quality in breakfast skippers, but sleep duration is not affected. The only study that measured sleep objectively in adults with sleep apnea reported higher wake after sleep onset (WASO) in breakfast skippers relative to breakfast eaters [24]. The only interventional study showed that eating a high-protein breakfast, compared with skipping breakfast, improved self-reported sleep quality, but not sleep efficiency, measured by accelerometry.

#### 3.2.2. Late eating

**Fig. 3.**
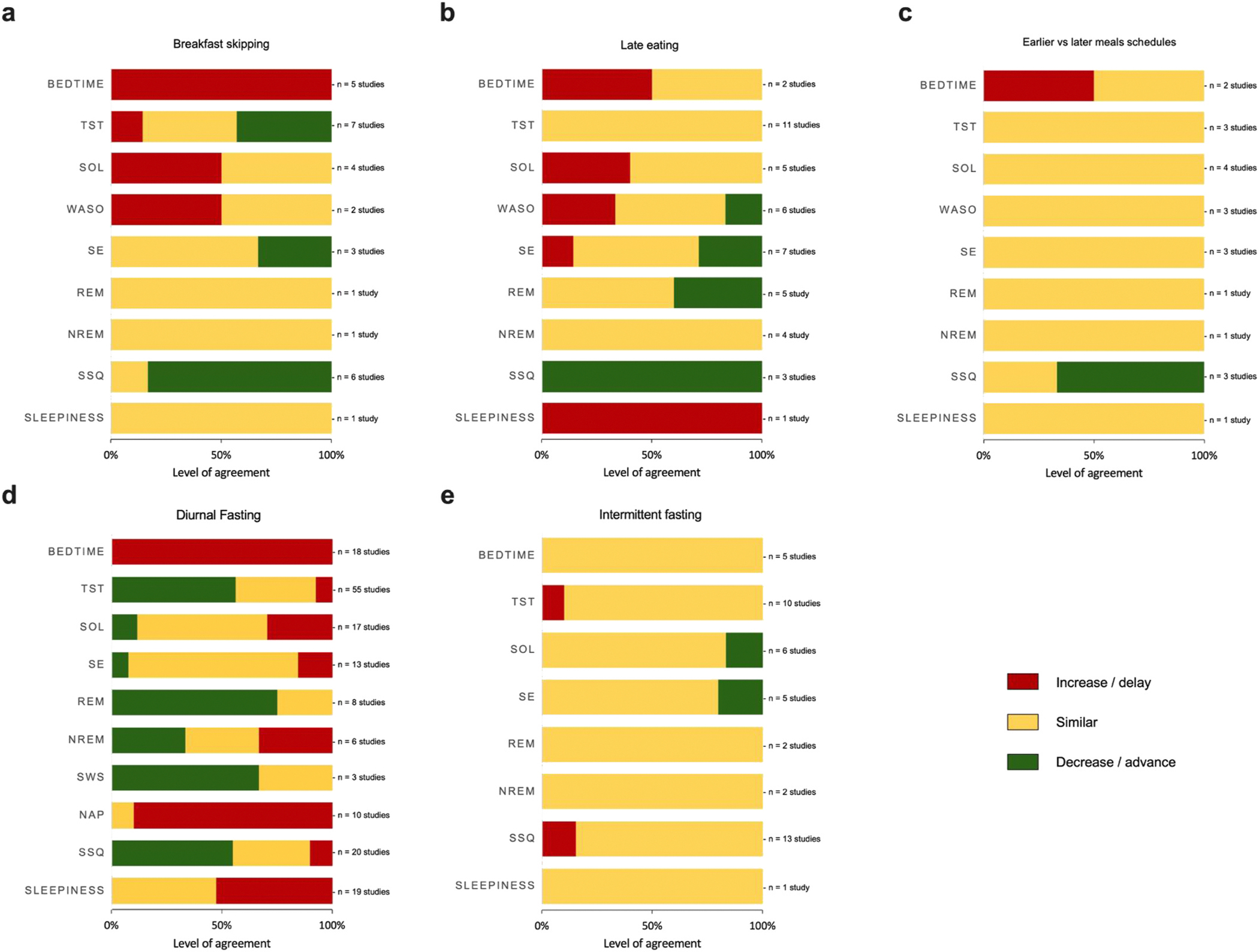
Agreement of studies on the effect of chrono-nutrition sub-dimensions on sleep outcomes. TST: total sleep time, SOL: sleep onset latency, WASO: wake after sleep onset, SE: sleep efficiency, REM: rapid-eye movement sleep, NREM: non-rapid-eye movement sleep, SSQ: Subjective sleep quality, SWS: slow wave sleep. * A coding scheme was elaborated in order to allow a classification of the level of agreement between the existing studies using the traffic light approach. Yellow color indicates the proportion of studies reporting no effect for each sleep variable, while green color indicates an increase and red a decrease. For bedtime, green color indicates an advance where red color indicates a delay. (For interpretation of the references to color in this figure legend, the reader is referred to the Web version of this article.)

Several studies examined the effect of late eating on sleep (n = 16) (Fig. S1b), of which five used objective sleep measures, five used self-report measures, and six used mixed methods. Only three studies included pediatric populations. Eleven studies were observational and five studies were experimental. As shown in Fig. 3b, we found one to 11 studies per sleep outcome.

Two cross-sectional studies examined the effect of late eating on bedtime. One documented a delay in bedtime of approximately 30 min after late eating [25] while the other found no discernible association between late eating and bedtime [24]. There was no association between late eating and sleep duration among eight studies. Reid et al. (2014) reported a negative association between eating closer to bedtime and sleep duration [26]. From a prospective study (10-d follow-up) of professional athletes, Falkenberg et al. (2020) reported that a longer time between eating dinner and bedtime was associated with shorter sleep duration [27]. The majority of observational studies based on both self-report and polysomnographic assessment support a negative association between eating close to bedtime (dinner and late snacks) and sleep quality, and have repeatedly reported markers of altered sleep onset and continuity.

Interventional studies have reported conflicting results. Uçar et al. (2021) reported higher self-reported sleep disturbance scores after the slow-digesting meal (protein + fat-rich meal) compared to a late, easily digestible meal (starch + sugar-rich meal) or a control session (no food intake after 19:00 h) [28]. However, the order of the sessions was not randomized and dietary intake and patterns prior to arrival at the laboratory were not specified. Another study examined the effect of a late-night meal on gastroesophageal reflux and sleep in adults with heartburn symptoms [29]. No valuable differences in PSG-measured sleep parameters were found. These findings suggest that while late eating may lead to more reflux events immediately after eating, it does not necessarily interfere with sleep quality when participants retire to sleep 2 h after their last meal [29]. Driver et al. (1999) also reported no effect on sleep quality and stages, but an increase in nocturnal body temperature, in a laboratory RCT conducted among seven young adults [30]. Two experimental studies focused on delaying dinner time [31, 32]. Although intricated with the sub-dimension of earlier vs later meals schedules, we decided to include these studies in late eating sub-dimension given that only dinner time was manipulated, and that the meal-to-bedtime window was very short (60 and 90 min). The study by Duan et al. (2021) showed no immediate effect on conventional polysomnographic sleep outcomes [31]. However, they reported an increase in delta power band referring to deeper sleep during the first segment of the night. Lehmann et al. (2022). found improvement in sleep efficiency explained by reduced WASO after five days of late dinner compared to routine dinner time in young athletes [32].

#### 3.2.3. Earlier vs later meals schedules

Six studies assessed the associations of meals schedules with sleep (Fig. S1c), of which two evaluated sleep by objective methods, two by self-report methods, and two used both methods. All studies were carried out in adult populations.

Two cross-sectional studies published over the past year examined the effect of delayed meal schedules on sleep quality. Bazzani et al. (2022) included 1298 participants during the COVID-19 pandemic. Although no direct comparison of different meal timing was undertaken, it was reported that participants who exhibited poor subjective sleep quality (PSQI>5) preferred delaying their first and last meals more so than good sleepers according to a chrono-nutrition questionnaire [33]. Loo et al. (2022) found a negative association between poor sleep quality and later meals among 90 pregnant women [34].

By contrast, experimental studies examining the effects of early versus late eating patterns on sleep quality found no major effects on sleep. In a pilot study by Pizinger et al. (2018), sleep was assessed by both accelerometry and PSG in a laboratory setting in six participants of both sexes after three days of four conditions (normal or late sleep and normal or late eating) [35]. The late eating condition delayed all meal times by 3.5 h compared to the habitual meal timing routine. No effects on sleep were reported. Wehrens et al. (2017) induced a 5-h delay in all meal times (breakfast, lunch, and dinner) for six days among ten healthy adults [36]. This cross-over RCT standardized meal intake and prohibited physical exercise during the experimental protocol. They reported no effects on sleep parameters evaluated by accelerometry. In addition, there was no effect on sleepiness and melatonin. Ruddick-Collins et al. (2022) evaluated the effect of caloric distribution (morning energy load versus evening energy load) in the context of a four-week, calorie-restricted, isoenergetic weight loss program in 30 participants with overweight/obesity [37]. This study found no clinically significant differences in sleep duration or quality between the two dietary conditions. Only one cross-over study conducted over a longer period (eight weeks) with ecological settings reported a delay of sleep onset latency (SOL) by 25 min with the late eating window compared to the early eating window condition (08:00 h–19:00 h vs. 12:00 h–23:00 h) [38]. No other effects on sleep duration or quality were observed.

### 3.3. Irregular meal pattern

#### 3.3.1. Diurnal fasting

Several studies evaluating the effect of diurnal fasting on sleep were identified (n = 65), of which 53 were observational and 12 were experimental (Fig. 3d). Forty-nine studies evaluated the quality and/or duration of sleep by self-report methods, ten by objective methods, and six used both methods (Fig. S2a). Studies were mainly conducted among adults (n = 63). All included diurnal fasting studies focused on Ramadan fasting.

Overall, the studies reported a change in sleep patterns during diurnal fasting with a delay in bedtime and possible reduction in nocturnal sleep duration. The latter was reported in 31 studies. The other four studies reported an increase, and 20 studies reported similar sleep duration. However, it is important to highlight an increase in nap duration reported in nine out of ten studies. Although the majority of studies investigating self-reported sleep quality showed a decrease in sleep quality (n = 11 out of 20), seven studies reported no effect and two reported an improvement. The results for markers of sleep quality from objective methods are less conclusive. The majority of studies reported no effect on SOL or sleep efficiency (SE). Analysis of sleep stages showed a possible decrease in rapid-eye movement (REM) sleep as reported by six out of eight studies. Although the effect of diurnal fasting on non-REM (NREM) sleep was less conclusive, two out of three studies reported a decrease in slow wave sleep (SWS). Moreover, there was a trend toward increased daytime sleepiness (n = 10). However, nine studies reported no effect of diurnal on sleepiness.

#### 3.3.2. Intermittent fasting

Intermittent fasting has previously been defined as the consumption of no or minimal amounts of calories during a fast that is interrupted by hours to days of usual levels of caloric intake [39]. Sixteen studies were included that focused on either eating windows (one observational study) or different fasting regimens (n = 15 interventional studies) (Fig. 3e).

The cross-sectional study found that eating window was associated with sleep time where adults with an eating duration ≤12 h reported longer sleep duration than those with an eating duration >12 h [24]. Adults with a longer eating window also had higher energy and carbohydrate intakes.

Of 15 studies of experimentally-induced intermittent fasting, nine evaluated the quality and/or duration of sleep by self-report methods, two by objective methods, and four used mixed methods (Fig. S2b). Studies were exclusively conducted in adults. Twelve of the included studies investigated time-restricted eating (TRE), two studies focused on alternate-day fasting (ADF), and one examined the effect of seven days of complete fasting on sleep. Sleep duration was examined in ten studies and self-reported sleep quality was reported in 13. Ten of the TRE studies found no effect on sleep while two found improvements. Self-reported sleep quality improved in one study [40] while SOL was reduced and SE increased in the other [41]. However, in the latter study, TRE was combined with an ADF regimen. Conversely, the other two studies examining the effect of ADF found no discernible effects on either sleep duration or self-reported sleep quality. Finally, an early study focusing on seven days of complete fasting reported an increase in sleep duration and self-reported sleep quality [42]. However, PSG sleep parameters in this study did not change.

#### 3.3.3. Day-to-day meal patterns

In this sub-dimension, three observational studies found adverse effects of irregular daily meal patterns on sleep quality. Beigrezaei et al. (2022) reported higher odds of insomnia in adolescent girls with irregular meal patterns [43]. Faris et al. (2021) found that irregular mealtimes were correlated with poor self-reported sleep quality in university students [44]. In addition, Tahara et al. (2021) reported that sleep problems were associated with irregular daily meal patterns in a cross-sectional study of up to 4490 Japanese workers [45].

### 3.4. Meal frequency

Only three studies examined the effect of meal frequency on sleep, two using subjective methods and one using mixed methods. In medical students, eating two meals or less per day was associated with later wake time, and eating three meals or more was associated with better self-reported sleep quality [46]. In contrast, in adolescent girls, meal frequency was not associated with insomnia symptoms [43]. Finally, one study found no association between meal frequency at baseline and sleep duration or bedtime seven days later [26]. However, qualitative sleep parameters were not assessed in this study.

## 4. Discussion

In recent years, the effect of the temporal characteristics of eating on sleep has gained significant prominence with a substantial surge in studies published over the past six years. To isolate the potential effects of exposure to each observed or experimentally induced temporal dietary phenomenon on sleep, we categorized the existing literature into dimensions and sub-dimensions providing a synthetic and illustrative framework. In the following sections, we discuss the findings from each chrono-nutrition sub-dimension, shedding light on current literature limitations, perspectives, and, where available, potential underlying mechanisms.

### 4.1. Meal timing

#### 4.1.1. Breakfast skipping

The majority of findings in this section are correlational and preclude establishing a causal relationship between skipping breakfast and sleep. Nonetheless, consistent studies endorse the idea that skipping breakfast is linked to delayed bedtime. Going to bed late is often associated with waking up late, which may interfere with social pressures and hinder breakfast consumption. The association between skipping breakfast and reduced sleep duration was found in youth but not adults. A potential explanation for these varying outcomes may be attributed to later bedtimes in youth compared to adults [47]. The only study using an experimental approach compared skipping breakfast vs. consuming a high-protein breakfast. Although self-reported measures underlined better sleep outcomes following the high-protein breakfast condition, this was not supported by the objective measure [48]. However, the limited number of participants and modest sensor sensitivity used in this study may have influenced the results. Thus, more interventional studies using PSG with robust designs and larger sample sizes are urgently needed to gain a more complete understanding of the causal effects of skipping breakfast on sleep quality and staging. Additional research is needed to investigate whether skipping breakfast leads to subsequent meal delays under real-life conditions, especially since some studies suggest that skipping breakfast is associated with late eating [49]. It is imperative to determine whether breakfast skipping in ecological contexts merely induces a delay in meal intake without narrowing the eating window, or whether it also initiates a pattern similar to TRE. We highlight the interconnected nature of these two chrono-nutrition paradigms. Future research is warranted to disentangle the effects of breakfast skipping from those of TRE because their effects on metabolism and sleep could be different. Furthermore, it is noteworthy that skipping breakfast has a discernible effect on subsequent meal content. Studies have reported an increase in caloric intake at both lunch and dinner [50]. In addition, this practice is associated with reduced diet quality, including lower fruit and dairy consumption and higher intake of refined grains [51]. Therefore, it’s important to understand whether the observed effects on sleep are due to breakfast skipping *per se* or to the effect of breakfast skipping on subsequent dietary intake timing and content.

#### 4.1.2. Late eating

Despite the absence of discernible impact on sleep duration, some studies have reported adverse effects on sleep quality. The conflicting findings may stem from variations in protocols, meal-to-bedtime interval, participant characteristics, meal compositions, and methodologies used to assess sleep.

We emphasize the critical role played by the meal-to-bedtime interval. The digestive mechanisms and body temperature increase may be similar regardless of the timing of the last meal. However, variations in the kinetics of these mechanisms could occur and disrupt sleep if the time lapse between the last meal and bedtime is short. In addition, elevated core body temperature may persist beyond gastric emptying, which depends on the nutrient composition of the consumed meal. These physiological processes, coupled with increased cortisol levels, postprandial hyperglycemia, hyperlipemia and, in some cases, increased oxidative stress, could affect sleep. In fact, there is a close relationship between sleep and the hypothalamic-pituitary-adrenal (HPA) axis. Sleep onset typically corresponds to decreased activity in the HPA axis. However, it is well known that cortisol levels rise after the consumption of a meal and remain elevated for 2–3 h depending on the nutrient composition [52]. Surprisingly, experimental studies in healthy adults or athletes reported no effect [29,30] or even improvement [31,32] in sleep after a late meal consumed 2 h before bedtime. Since gastric emptying normally takes 90–120 min [53], we can assume that this process would have been attenuated before bedtime limiting the interference with sleep. Therefore, further examination of the effect of shorter meal-to-bedtime interval on sleep is needed.

Conversely, Duan et al. (2021) suggested that postprandial sleepiness may help to achieve a deeper sleep state more efficiently [31]. Postprandial sleepiness stems from an inherent human trait inherited from our hunting and foraging ancestors. This tendency may be rooted in the theory that humans are wired to stay alert when hungry, to promote food-seeking behaviors, and, once food is secured and consumed, the tendency is to relax and rest. In fact, during prolonged periods of low energy availability, neuronal populations (mainly orexin) involved in sleep-wake and metabolic pathways may favor arousal over sleep for foraging and survival purposes. Consequently, an excessively long window between the last meal and bedtime could also activate orexigenic signals in the brain, promoting wakefulness and potentially leading to fragmented sleep and early awakenings [54]. Falkenberg et al. (2021) observed an inverse association between length of the last meal-to-bedtime window and sleep duration in professional athletes [27]. This was endorsed by the results of Lehmann et al. (2022) comparing routine dinner (210 min before bedtime) and late dinner (90 min before bedtime) effects on sleep in young elite rugby players [32]. Decreases in WASO and microarousals were highlighted. The authors argued that dinner must provide sufficient energy to ensure appropriate body and brain functioning for the night fasting period. Athletes may therefore present difficulty maintaining sleep if the time elapsed between dinner and sleep is too long. However, these results may not be transferable to the general population because the effect of late dinner on sleep continuity might be different in athletes, who have high energy demands, enhanced metabolic flexibility and glucose uptake by skeletal muscles [55]. Although a late meal might enhance sleep quality among athletes, individuals with obesity and/or metabolic impairment might not experience the same effect. Research suggests that these individuals often show hyperglycemia, changes in dynamics of antioxidant response, and increased inflammation after consuming a meal or undergoing a glucose tolerance test, compared to their healthy counterparts [56,57]. A recent review suggested that postprandial hyperglycemia resulting in compensatory hyperinsulinemia may lead to mild reactive hypoglycemia, which affects the activity of orexin neurons and results in sleep disturbances [58]. Finally, while the impact on sleep of the last eating occasion remains uncertain based on current research, it is important to emphasize that late eating aligns with biologically unfavorable times for energy and macronutrient metabolism [59]. A substantial body of research underscores that such late eating patterns can induce circadian misalignment, potentially leading to increased body weight and increased cardiometabolic risk.

In summary, the last meal appears to be intertwined with various physiological and neurobiological processes influencing sleep. However, given the mixed results, further studies that carefully control for potential confounding factors (i.e., content of the last meal, meal timing, bedtime, content and frequency of the preceding meals) are needed to provide accurate recommendations. We emphasize the importance of controlling meal content, as gastric emptying, induced thermogenesis, cortisol levels, and metabolic responses are largely influenced by meal composition. Moreover, particular attention should be given to participant characteristics, such as level of physical activity and cardiometabolic status. Finally, research investigating the effects of the last meal-to-bedtime window on pathways such as digestive processes, postprandial hyperglycemia, appetite, hormonal, orexigenic, and neurotransmitter signaling is critical. Such comprehensive efforts will contribute to evidence-based guidelines regarding the optimal meal-to-bedtime window.

#### 4.1.3. Earlier vs later meals schedules

Conflicting results emerge when the results of experimental studies that shift meal times are juxtaposed with the results of cross-sectional studies or those from other sub-dimensions of meal timing. All studies testing delays in meal schedules or comparing early versus late energy load in the short term found no impact on sleep. Only one study maintaining early versus late eating window for eight weeks in ecological settings found delayed bedtime with no effects on sleep quality.

There are several key points that may explain the lack of effects observed here. First, the current sub-dimension includes robust and well-controlled experimental studies. Energy intake and meal composition were rigorously matched. In addition, physical activity was measured and participants were prohibited from structured exercise. This may suggest that the magnitude of the effects of skipping breakfast or late eating observed in the previous sections may be due to differences in food quantity and quality or physical activity levels of participants. However, most studies from this sub-dimension focused on metabolism and included sleep as a secondary outcome. Only one pilot study used PSG in a limited number of participants [35], while the remaining studies used self-reported methods or accelerometry which may not be sensitive enough to detect differences in sleep, especially in the short term [36–38]. Therefore, further experimental studies comparing the effect of an early versus late eating window on sleep using gold standard measures are eagerly awaited.

### 4.2. Irregular meal pattern

#### 4.2.1. Diurnal fasting

Overall, this sub-dimension highlights some adverse effects of diurnal fasting on sleep, namely a tendency toward a decrease in nocturnal sleep duration. However, including nap duration in the calculation of total sleep time could potentially mitigate the observed effects and result in a net increase in sleep duration in some studies. The findings also reported shifts in sleep schedules, with delays in both bed and wake times during Ramadan. These changes in sleep schedules often culminate in a reduction in the duration of nighttime sleep and may account for the observed decrease in REM sleep as well as increased daytime sleepiness.

The majority of studies included in this sub-dimension were based on observations of Ramadan fasting, in which individuals engage in diurnal intermittent fasting, abstaining from food and beverages from sunrise to sunset, suggesting consumption of meal at the beginning and the end of the rest biological phase. Ramadan follows the Arabic calendar, which is governed by the lunar system. This peculiarity of the calendar causes Ramadan to occur in different seasons, thus affecting the length of the fasting period, which depends on the varying lengths of daylight which is subject to geographical location. The inconsistency in the results may be due in part to the different assessment times (first, second, third, or last week of Ramadan) and length and timing of fasting according to daylight (i.e. season and location). Some of this information was not systematically reported across the included studies and should be explicitly addressed in future research.

Although Ramadan fasting was classified as an irregular eating dimension [60], the specifics of this practice make it difficult to isolate the effects of the temporal changes in food intake from those of other confounding factors. The Ramadan period is generally associated with reduced daytime activities and increased nighttime light exposure [61] and nocturnal religious or social traditions. Bahamman et al. (2005) reported that bedtime and wake time were also affected in non-fasting participants [62], suggesting that the effect on sleep schedules may not be an exclusive consequence of eating patterns during Ramadan. In addition, it’s worth noting that adherence to Ramadan fasting may vary among individuals for a variety of reasons, including cultural practices and personal preferences. For example, not all Ramadan fasters contend with two meals (the breaking of the fasting meal “Iftar” and the pre-dawn meal “Suhoor”) and some may consume several meals during the night. In particular, some fasters may go back to sleep following the Suhoor meal. Furthermore, previous studies have reported increased fat intake and a shift in carbohydrate intake from complex to more simple sugars, along with possible evening consumption of caffeinated beverages [63]. Some experimental studies that controlled for environmental conditions and sleep-wake patterns during Ramadan fasting did not find changes in sleep quality and staging [64,65]. However, to overcome these confounding factors, the effect of diurnal fasting needs to be studied outside of the context of Ramadan.

#### 4.2.2. Intermittent fasting

While the average eating window in modern society may be > 14 h [66–69], the majority of studies included in this sub-dimension did not report discernible effects on sleep. However, few studies measured sleep objectively and controlled for energy intake [41]. Most of studies were designed to assess the effect of TRE on weight loss, and some even induced a caloric restriction concomitant with the TRE condition. We also note the high heterogeneity in study protocols with eating windows ranging from 4 h per day [70] to 10 h [71]. In addition, some studies imposed specific meal times, while others relied on self-selected eating occasions and times, sometimes with no information on day-to-day consistency. Finally, some studies omitted meals, thereby manipulating eating frequency during their TRE intervention. Only one TRE study used PSG and found no effect on sleep [72]. However, this study also imposed a calorie restricted ketogenic diet (1350 kcal) with two eating occasions. Future studies testing the effect of the TRE regimen *per se* on objectively measured sleep should address these shortcomings. Further research comparing the effects of daytime and nighttime fasting on sleep should be conducted, accompanied by efforts to investigate explanatory pathways.

Given the few studies focusing on complete fasting, as well as the mixed and subjective nature of the findings, no consensus can be reached for the moment and further studies using objective methods are needed.

#### 4.2.3. Day-to-day meal pattern

Irregular daily eating patterns was consistently associated with poor self-reported sleep quality in observational studies. Studies with experimental designs are now needed to test causality.

### 4.3. Meal frequency

Emerging evidence suggests that lower than three meal frequency may lead to metabolic dysfunction and have adverse effects on glucose levels and insulin sensitivity [73]. Previous research has found an association between glycemic variability in people with type 1 diabetes and objectively assessed sleep quality [74]. Surprisingly, the effect of glycemic variability on sleep in healthy populations remains an unexplored area of research. While the hypothesis of a potential effect of meal frequency on glucose metabolism and sleep is an intriguing avenue of investigation, this hypothesis remains speculative due to the paucity of research.

### 4.4. Limitation, gaps, and perspectives

One of the most important limitations in this field is the heterogeneity of the literature in terms of study design and population characteristics which did not allow clear conclusions. Future chrono-nutrition research should report details on seasonality, as this information is essential for the interpretation of the results. Further research on chrono-nutrition and sleep is awaited, particularly experimental studies, as research is needed to elucidate the underlying mechanisms involved in this interaction.

The physiological response to chrono-nutrition is subject to variability based on participant characteristics such as metabolic health, chronotype, and physical activity level. Thus, it is imperative to conduct studies that compare the effects of chrono-nutrition on sleep outcomes in healthy individuals with those who suffer from insomnia, other sleep disorders, or chronic conditions such as obesity and metabolic disorders. In addition, elucidating the role of chronotype in mediating the relationship between chrono-nutrition and sleep is critical for future research directions. Chronotype is a circadian phenotype that is indicative of the acquired circadian clock system of an individual [75]. It is hypothesized that chronotype may modulate the effects of environmental synchronizers on sleep. For example, results from a recent study suggest that the timing of exercise influences sleep outcomes in athletes depending on their chronotype [76]. It remains to be investigated whether similar chronotype-dependent effects exist in chrono-nutrition interventions. In addition, the consideration of physical activity levels and timing in chrono-nutrition studies is essential. Physical activity may not only directly influence sleep [77], but may also interact with the effects of chrono-nutrition on sleep, thereby introducing potential confounding variables. Therefore, future studies should carefully consider and control for physical activity variables to better understand the nuanced relationship between chrono-nutrition, physical activity, and sleep.

The existing definition of chrono-nutrition often fails to delineate an exposure or intervention that isolates a specific temporal aspect of dietary patterns for investigation. The complexity and intricacy of certain sub-dimensions and the lack of crucial detailed information on exposures or interventions typically result in significant heterogeneity, which poses a significant challenge to the comparability of study results and prevents the conduct of systematic reviews and meta-analyses. Careful examination of the actual definition of chrono-nutrition raises inevitable questions. For example, can we consider skipping breakfast as a TRE? When do we consider a meal intake as late? Can we consider Ramadan fasting in summer as a comparable exposure to Ramadan fasting in winter? Furthermore, TRE mainly involves a shorter eating window but this may also implicate alterations in timing and frequency of meals. Some TRE studies may advance meal schedules compared to habitual patterns, while others introduce a delay. In addition, some studies controlled for day-to-day variability while others did not. Therefore, there is an urgent need for a chrono-nutrition framework and consensus on the definition of all temporal variables related to eating patterns.

Second, future studies should standardize energy intake and meal content when examining the effect of chrono-nutrition. Parr et al. (2022) highlighted these limitations in a recent perspective and suggested the importance of integrating the “what with the when” [78], as this could be a major confounding factor in this area of research. Thus, they proposed a hypothetical synergy between the two factors (temporal characteristics of eating patterns and dietary content) that cannot be understood in light of the existing literature. For example, is the effect of skipping breakfast on sleep solely a chrono-nutrition effect if it involves differences in subsequent food intake content? Moreover, should we consider the impact of consuming a late high-protein meal as similar to consuming a late high-carbohydrate meal? Thus, future studies should disentangle the effects of chrono-nutrition *per se* from those of the downstream effects on subsequent dietary intake. Also, the effect of chrono-nutrition manipulation along with different meal compositions (high fat, high carbohydrate, high protein) on sleep should be investigated.

## 5. Conclusion

Modern lifestyles, shaped by technological advances, and unrestricted access to foods, have brought about significant shifts in eating patterns which could influence sleep health. Overall, this review highlights important evidence linking the temporal characteristics of eating patterns to various facets of sleep health. However, additional research is needed to inform chrono-nutrition guidelines to improve sleep health in the general population.

## Supplementary Material

Supportive file B

Supportive file A

## Practice points

- Breakfast skipping is associated with delayed bedtime and lower self-reported sleep quality.
- Breakfast skipping is associated with shorter sleep duration in youth but not adults.

## Research agenda

- RCTs with robust sleep measures are needed.
- New research should disentangle the effects of breakfast skipping *per se* from those of the downstream effects on subsequent dietary intake.

## Practice points

- Limited evidence reported differences in sleep duration following late eating.
- Most of the observational studies reported poor sleep quality following late eating.
- Experimental studies were inconsistent regarding the effect of late eating on objectively-measured sleep quality.
- The timing of late meals, meal nutrient content, and study populations varied between studies, preventing a consensus on the effect on sleep.

## Research agenda

- Last meal-to-bedtime window could be used as a marker to assess the effect of late eating on sleep in order to provide future recommendations.
- Future studies should systematically screen enrolled subjects for eating disorders (binge eating, night eating syndrome).
- The effect of last meal-to-bedtime window on digestive processes, hormonal, orexigenic, and neurotransmitter signals should be investigated in future studies.
- The effect of different last meal compositions (high fat, high carbohydrate, high protein) on sleep should be investigated in future studies.

## Practice points

- Experimental studies comparing early versus late meal times in the short term found no differences in sleep outcomes.

## Research agenda

- Future studies with sleep as a primary outcome, using rigorous objective and self-reported sleep measures, are needed.

## Practice points

- Ramadan fasting can lead to adverse effects on sleep patterns, including a decrease in nocturnal sleep duration, delay in sleep schedules, reduced REM sleep, and increased daytime sleepiness but confounding from other factors is problematic (lifestyle changes, nighttime religious or social traditions, differences in social pressures, and changes in dietary intake).
- Experimental studies that controlled for environmental conditions and sleep-wake patterns during Ramadan fasting did not find changes in sleep quality and staging.

## Research agenda

- Diurnal fasting effects on sleep need to be studied beyond the context of Ramadan.

## Practice points

- Most studies did not report significant effects of intermittent fasting on sleep, but conclusions are challenged by heterogeneity in study protocols.

## Research agenda

- Future studies should examine the empirical effect of TRE on sleep.
- Future studies should compare early vs late TRE effect on objective sleep.
- Further studies examining the effect of alternate-day fasting and complete fasting on sleep are needed.

## Abbreviations

ADF: alternate-day fasting
HPA: hypothalamic-pituitary-adrenal
NREM: non-REM sleep
PSG: polysomnography
REM: rapid eye movement sleep
SE: sleep efficiency
SOL: sleep onset latency
SWS: slow wave sleep
TRE: time-restricted eating
WASO: wake after sleep onset
