# Supplementary material for: Chrono-nutrition and sleep: lessons from the temporal feature of eating patterns in human studies - A systematic scoping review
Source: Sleep Med Rev. Author manuscript; Available in PMC 2025 Aug 1. (PMC12090848; doi:10.1016/j.smrv.2024.101953)
Supplement: Supportive file B [file NIHMS2076074-supplement-Supportive_file_B.docx]

**Chrono-nutrition and sleep - lessons from the temporal feature of eating patterns in human studies: A systematic scoping review**

**Authors**

Oussama Saidi ^1*^, Emmanuelle Rochette^1,2,3^, Lou Dambel ^1^, Marie-Pierre St-Onge ^4^, Pascale Duché ^1^

**Affiliations**

1. JAP2S laboratory, Toulon University, F-83041 Toulon, France.
2. Department of Pediatrics, Clermont-Ferrand University Hospital, F-63000 Clermont-Ferrand, France
3. Clermont Auvergne University, INSERM, CIC 1405, CRECHE unit, F-63000 Clermont-Ferrand, France
4. Center of Excellence for Sleep and Circadian Research and Division of General Medicine, Department of Medicine, Columbia, University Irving Medical Center, New York, NY, USA 10032.

**OCRID IDs**

Oussama Saidi: 0000-0003-3005-8716

Emmanuelle Rochette: 0000-0001-5180-9916

Marie-Pierre St-Onge : 0000-0003-1354-1749

Pascale Duché: 0000-0001-7206-6429

***Corresponding author**

Dr. Oussama SAIDI, IAPS Laboratory, Toulon University, F-83041 Toulon, France

E-mail: oussama.saidi@univ-tln.fr

Tel: +33 6 58 48 72 88

**Financial disclosure:**

Marie-Pierre St-Onge is funded in part by the National Institutes of Health grants R01 DK128154, R01 HL142648 and R35 HL155670

Supporting File B (online supplement)

**References of included studies according to chrono-nutrition dimensions and sub-dimensions**

**Meal Timing**

- ***Breakfast skipping:***

- Azemati B, Heshmat R, Qorbani M, Ahadi Z, Azemati A, Shafiee G, et al. Association of meal skipping with subjective health complaints in children and adolescents: the CASPIAN-V study. Eating and Weight Disorders-Studies on Anorexia, Bulimia and Obesity 2020;25:241–6.

Beigrezaei S, Mazidi M, Davies IG, Salehi-Abargouei A, Ghayour-Mobarhan M, Khayyatzadeh SS. The association between dietary behaviors and insomnia among adolescent girls in Iran. Sleep Health 2022;8:195–9.

Faris ME, Vitiello MV, Abdelrahim DN, Cheikh Ismail L, Jahrami HA, Khaleel S, et al. Eating habits are associated with subjective sleep quality outcomes among university students: findings of a cross-sectional study. Sleep and Breathing 2021:1–12.

Gwin JA, Leidy HJ. Breakfast consumption augments appetite, eating behavior, and exploratory markers of sleep quality compared with skipping breakfast in healthy young adults. Current Developments in Nutrition 2018;2:nzy074.

Lebacq T, Holmberg E, Pedroni C, Dujeu M, Castetbon K. Weekday sleep duration and morning tiredness are independent covariates of breakfast skipping in adolescents. European Journal of Clinical Nutrition 2022;76:1403–8.

Liu A, Fan J, Ding C, Yuan F, Gong W, Zhang Y, et al. The association of sleep duration with breakfast patterns and snack behaviors among Chinese children aged 6 to 17 years: Chinese National Nutrition and Health Surveillance 2010–2012. Nutrients 2022;14:2247.

Lopes T do VC, Borba ME, Lopes R do VC, Fisberg RM, Lemos Paim S, Vasconcelos Teodoro V, et al. Eating late negatively affects sleep pattern and apnea severity in individuals with sleep apnea. Journal of Clinical Sleep Medicine 2019;15:383–92.

Manmee C, Tanavikrankoon M, Janpol K. Breakfast Skipping among Personnel in Rajavithi Hospital. Journal of the Medical Association of Thailand= Chotmaihet Thangphaet 2017;100:S222-9.

Reutrakul S, Hood MM, Crowley SJ, Morgan MK, Teodori M, Knutson KL. The relationship between breakfast skipping, chronotype, and glycemic control in type 2 diabetes. Chronobiology International 2014;31:64–71.

Tambalis KD, Panagiotakos DB, Psarra G, Sidossis LS. Breakfast skipping in Greek schoolchildren connected to an unhealthy lifestyle profile. Results from the National Action for Children’s Health program. Nutrition & Dietetics 2019;76:328–35.

Thivel D, Isacco L, Aucouturier J, Pereira B, Lazaar N, Ratel S, et al. Bedtime and sleep timing but not sleep duration are associated with eating habits in primary school children. Journal of Developmental & Behavioral Pediatrics 2015;36:158–65.

Yasuda J, Asako M, Arimitsu T, Fujita S. Skipping breakfast is associated with lower fat-free mass in healthy young subjects: a cross-sectional study. Nutrition Research 2018;60:26–32.

Zhou Y, Bo S, Ruan S, Dai Q, Tian Y, Shi X. Deteriorated sleep quality and influencing factors among undergraduates in northern Guizhou, China. PeerJ 2022;10:e13833.

- ***Late eating:***

Chung N, Bin YS, Cistulli PA, Chow CM. Does the Proximity of Meals to Bedtime Influence the Sleep of Young Adults? A Cross-Sectional Survey of University Students. Int J Environ Res Public Health 2020;17:E2677. https://doi.org/10.3390/ijerph17082677.

Crispim CA, Zimberg IZ, dos Reis BG, Diniz RM, Tufik S, de Mello MT. Relationship between food intake and sleep pattern in healthy individuals. Journal of Clinical Sleep Medicine 2011;7:659–64.

Driver HS, Shulman I, Baker FC, Buffenstein R. Energy content of the evening meal alters nocturnal body temperature but not sleep. Physiology & Behavior 1999;68:17–23.

Duan D, Gu C, Polotsky VY, Jun JC, Pham LV. Effects of Dinner Timing on Sleep Stage Distribution and EEG Power Spectrum in Healthy Volunteers. Nat Sci Sleep 2021;13:601–12. https://doi.org/10.2147/NSS.S301113.

Falkenberg E, Aisbett B, Lastella M, Roberts S, Condo D. Nutrient intake, meal timing and sleep in elite male Australian football players. Journal of Science and Medicine in Sport 2020.

Faris ME, Vitiello MV, Abdelrahim DN, Cheikh Ismail L, Jahrami HA, Khaleel S, et al. Eating habits are associated with subjective sleep quality outcomes among university students: findings of a cross-sectional study. Sleep and Breathing 2021:1–12.

Garaulet M, Gómez-Abellán P, Alburquerque-Béjar JJ, Lee Y-C, Ordovás JM, Scheer FAJL. Timing of food intake predicts weight loss effectiveness. Int J Obes (Lond) 2013;37:604–11. https://doi.org/10.1038/ijo.2012.229.

Hermenegildo-López Y, Donat-Vargas C, Sandoval-Insausti H, Moreno-Franco B, Rodríguez-Ayala M, Rey-García J, et al. A higher intake of energy at dinner is associated with incident metabolic syndrome: A prospective cohort study in older adults. Nutrients 2021;13:3035.

Lehmann L, Saidi O, Giacomoni M, Del Sordo G, Maso F, Margaritis I, et al. A Delayed Evening Meal Enhances Sleep Quality in Young Rugby Players. International Journal of Sport Nutrition and Exercise Metabolism 2022;33:39–46.

Lopes T do VC, Borba ME, Lopes R do VC, Fisberg RM, Lemos Paim S, Vasconcelos Teodoro V, et al. Eating late negatively affects sleep pattern and apnea severity in individuals with sleep apnea. Journal of Clinical Sleep Medicine 2019;15:383–92.

Martínez-Lozano N, Tvarijonaviciute A, Ríos R, Barón I, Scheer FA, Garaulet M. Late eating is associated with obesity, inflammatory markers and circadian-related disturbances in school-aged children. Nutrients 2020;12:2881.

Orr, Harnish. Sleep‐related gastro‐oesophageal reflux: provocation with a late evening meal and treatment with acid suppression. Alimentary Pharmacology & Therapeutics 1998;12:1033–8.

Reid KJ, Baron KG, Zee PC. Meal timing influences daily caloric intake in healthy adults. Nutrition Research 2014;34:930–5.

Saidi O, Rochette E, Del Sordo G, Doré É, Merlin É, Walrand S, et al. Eucaloric Balanced Diet Improved Objective Sleep in Adolescents with Obesity. Nutrients 2021;13:3550.

Soreca I, Wallace ML, Hall MH, Hasler BP, Frank E, Kupfer DJ. The association between meal timing and frequency with cardiometabolic profile in patients with bipolar disorder. Acta Psychiatr Scand 2016;133:453–8. https://doi.org/10.1111/acps.12578.

Uçar C, Özgöçer T, Yıldız S. Effects of late-night eating of easily-or slowly-digestible meals on sleep, hypothalamo-pituitary-adrenal axis, and autonomic nervous system in healthy young males. Stress Health 2021;37:640–9. <https://doi.org/10.1002/smi.3025>.

- ***Earlier vs. later meals schedules***

Allison KC, Hopkins CM, Ruggieri M, Spaeth AM, Ahima RS, Zhang Z, et al. Prolonged, Controlled Daytime versus Delayed Eating Impacts Weight and Metabolism. Curr Biol 2021;31:650-657.e3. <https://doi.org/10.1016/j.cub.2020.10.092>.

Bazzani A, Marantonio S, Andreozzi G, Lorenzoni V, Bruno S, Cruz-Sanabria F, et al. Late chronotypes, late mealtimes. Chrononutrition and sleep habits during the COVID-19 lockdown in Italy. Appetite 2022;172:105951.

Loo RSX, Yap F, Ku CW, Cheung YB, Tan KH, Chan JKY, et al. Maternal meal irregularities during pregnancy and lifestyle correlates. Appetite 2022;168:105747.

Pizinger T, Kovtun K, RoyChoudhury A, Laferrère B, Shechter A, St-Onge M-P. Pilot study of sleep and meal timing effects, independent of sleep duration and food intake, on insulin sensitivity in healthy individuals. Sleep Health 2018;4:33–9.

Ruddick-Collins LC, Morgan PJ, Fyfe CL, Filipe JA, Horgan GW, Westerterp KR, et al. Timing of daily calorie loading affects appetite and hunger responses without changes in energy metabolism in healthy subjects with obesity. Cell Metabolism 2022;34:1472-1485. e6.

Wehrens SM, Christou S, Isherwood C, Middleton B, Gibbs MA, Archer SN, et al. Meal timing regulates the human circadian system. Current Biology 2017;27:1768-1775. e3.

**Irregular eating patterns**

- ***Religious fasting***

Abdul Razzak R, Mohamed MW, Alshaiji AF, Qareeballa AA, Bagust J, Docherty S. Is spatial orientation affected by Ramadan fasting? Nutrition and Food Science 2019;49:464–75. https://doi.org/10.1108/NFS-08-2018-0224.

Akbari HA, Yoosefi M, Pourabbas M, Weiss K, Knechtle B, Vancini RL, et al. Association of Ramadan participation with psychological parameters: A cross-sectional study during the COVID-19 pandemic in Iran. Journal of Clinical Medicine 2022;11:2346.

Al-Rawi N, Madkour M, Jahrami H, Salahat D, Alhasan F, BaHammam A, et al. Effect of diurnal intermittent fasting during Ramadan on ghrelin, leptin, melatonin, and cortisol levels among overweight and obese subjects: A prospective observational study. PLoS One 2020;15:e0237922. <https://doi.org/10.1371/journal.pone.0237922>.

Alghamdi AS, Alghamdi KA, Jenkins RO, Alghamdi MN, Haris PI. Impact of Ramadan on Physical Activity and Sleeping Patterns in Individuals with Type 2 Diabetes: The First Study Using Fitbit Device. Diabetes Therapy 2020;11:1331–46. <https://doi.org/10.1007/s13300-020-00825-x>.

Almeneessier AS, Bahammam AS, Sharif MM, Bahammam SA, Nashwan SZ, Perumal SRP, et al. The influence of intermittent fasting on the circadian pattern of melatonin while controlling for caloric intake, energy expenditure, light exposure, and sleep schedules: a preliminary report. Annals of Thoracic Medicine 2017;12:183.

Almeneessier AS, BaHammam AA, Alzoghaibi M, Olaish AH, Nashwan SZ, BaHammam AS. The effects of diurnal intermittent fasting on proinflammatory cytokine levels while controlling for sleep/wake pattern, meal composition and energy expenditure. PLoS One 2019;14:e0226034. https://doi.org/10.1371/journal.pone.0226034.

Almeneessier AS, BaHammam AA, Olaish AH, Pandi-Perumal SR, Manzar MD, BaHammam AS. Effects of Diurnal Intermittent Fasting on Daytime Sleepiness Reflected by EEG Absolute Power. J Clin Neurophysiol 2019;36:213–9. https://doi.org/10.1097/WNP.0000000000000569.

Alzhrani A, Alhussain MH, BaHammam AS. Changes in dietary intake, chronotype and sleep pattern upon Ramadan among healthy adults in Jeddah, Saudi Arabia: A prospective study. Frontiers in Nutrition 2022;9:966861.

Aziz AR, Wahid MF, Png W, Jesuvadian CV. Effects of Ramadan fasting on 60 min of endurance running performance in moderately trained men. Br J Sports Med 2010;44:516–21. https://doi.org/10.1136/bjsm.2009.070425.

Aziz AR, Che Muhamad AM, Roslan SR, Ghulam Mohamed N, Singh R, Chia MYH. Poorer intermittent sprints performance in Ramadan-fasted Muslim footballers despite controlling for pre-exercise dietary intake, sleep and training load. Sports 2017;5:4.

Aziz AR, Lim DSL, Sahrom S, Che Muhamed AM, Ihsan M, Girard O, et al. Effects of Ramadan fasting on match-related changes in skill performance in elite Muslim badminton players. Science and Sports 2020;35:308.e1-308.e10. https://doi.org/10.1016/j.scispo.2019.07.014.

BaHammam A. Sleep pattern, daytime sleepiness, and eating habits during the month of Ramadan. Sleep and Hypnosis 2003;5:165–74.

Bahammam A. Effect of fasting during Ramadan on sleep architecture, daytime sleepiness and sleep pattern. Sleep and Biological Rhythms 2004;2:135–43. https://doi.org/10.1111/j.1479-8425.2004.00135.x.

BaHammam A. Assessment of sleep patterns, daytime sleepiness, and chronotype during Ramadan in fasting and nonfasting individuals. Saudi Med J 2005;26:616–22.

BaHammam A, Alrajeh M, Albabtain M, Bahammam S, Sharif M. Circadian pattern of sleep, energy expenditure, and body temperature of young healthy men during the intermittent fasting of Ramadan. Appetite 2010;54:426–9. <https://doi.org/10.1016/j.appet.2010.01.011>.

Bahammam AS, Nashwan S, Hammad O, Sharif MM, Pandi-Perumal SR. Objective assessment of drowsiness and reaction time during intermittent Ramadan fasting in young men: a case-crossover study. Behav Brain Funct 2013;9:32. https://doi.org/10.1186/1744-9081-9-32.

BaHammam AS, Alaseem AM, Alzakri AA, Sharif MM. The effects of Ramadan fasting on sleep patterns and daytime sleepiness: An objective assessment. Journal of Research in Medical Sciences 2013;18:127–31.

Bahammam AS, Almushailhi K, Pandi-Perumal SR, Sharif MM. Intermittent fasting during Ramadan: does it affect sleep? J Sleep Res 2014;23:35–43. https://doi.org/10.1111/jsr.12076.

Bahammam A, Pandi-Perumal S, Alzoghaibi M. The effect of Ramadan intermittent fasting on lipid peroxidation in healthy young men while controlling for diet and sleep: A pilot study. Annals of Thoracic Medicine 2016;11:43–8. https://doi.org/10.4103/1817-1737.172296.

Bahijri S, Borai A, Ajabnoor G, Abdul Khaliq A, AlQassas I, Al-Shehri D, et al. Relative metabolic stability, but disrupted circadian cortisol secretion during the fasting month of Ramadan. PLoS One 2013;8:e60917. https://doi.org/10.1371/journal.pone.0060917.

Bener A, A Al-Hamaq AOA, Öztürk M, Çatan F, Haris PI, Rajput KU, et al. Effect of ramadan fasting on glycemic control and other essential variables in diabetic patients. Ann Afr Med 2018;17:196–202. https://doi.org/10.4103/aam.aam_63_17.

Bener A, Al-Hamaq AOAA, Öztürk M, Güllüoǧlu S. Does Ramadan fasting have effects on sleep, fatigue and blood pressure among patients with hypertension? Blood Pressure Monitoring 2021:108–12. https://doi.org/10.1097/MBP.0000000000000496.

Boujelbane MA, Trabelsi K, Jahrami HA, Masmoudi L, Ammar A, Khacharem A, et al. Time-restricted feeding and cognitive function in sedentary and physically active elderly individuals: Ramadan diurnal intermittent fasting as a model. Frontiers in Nutrition 2022;9:1041216.

Boukhris O, Hsouna H, Chtourou L, Abdesalem R, BenSalem S, Tahri N, et al. Effect of Ramadan fasting on feelings, dietary intake, rating of perceived exertion and repeated high intensity short-term maximal performance. Chronobiol Int 2019;36:1–10. https://doi.org/10.1080/07420528.2018.1513943.

Boukhris O, Hill DW, Ammar A, Trabelsi K, Hsouna H, Abdessalem R, et al. Longer nap duration during ramadan observance positively impacts 5-m shuttle run test performance performed in the afternoon. Frontiers in Physiology 2022;13:811435.

Bouzid MA, Abaïdia A-E, Bouchiba M, Ghattassi K, Daab W, Engel FA, et al. Effects of Ramadan Fasting on Recovery Following a Simulated Soccer Match in Professional Soccer Players: A Pilot Study. Frontiers in Physiology 2019;10. https://doi.org/10.3389/fphys.2019.01480.

Brini S, Castillo D, Raya-González J, Castagna C, Bouassida A, Khalifa R, et al. Basketball-Specific Small-Sided Games Training during Ramadan Intermitting Fasting: Do Changes in Body Composition, Sleep Habits, and Perceived Exertion Affect Technical Performance? Int J Environ Res Public Health 2021;18. https://doi.org/10.3390/ijerph182212008.

Brini S, Abderrahman AB, Clark CCT, Zouita S, Hackney AC, Govindasamy K, et al. Sex-specific effects of small-sided games in basketball on psychometric and physiological markers during Ramadan intermittent fasting: a pilot study. BMC Sports Science, Medicine and Rehabilitation 2021;13. <https://doi.org/10.1186/s13102-021-00285-1>.

Çelik Ö, Koçak T, Köksal E. Effects of diurnal ramadan intermittent fasting on cardiometabolic risk factors and sleep quality in healthy turkish adults. Ecology of Food and Nutrition 2022;61:595–607.

Chamari K, Haddad M, Wong DP, Dellal A, Chaouachi A. Injury rates in professional soccer players during Ramadan. J Sports Sci 2012;30 Suppl 1:S93-102. <https://doi.org/10.1080/02640414.2012.696674>.

Chamari K, Briki W, Farooq A, Patrick T, Belfekih T, Herrera CP. Impact of Ramadan intermittent fasting on cognitive function in trained cyclists: a pilot study. Biology of Sport 2016;33:49–56.

Chennaoui M, Desgorces F, Drogou C, Boudjemaa B, Tomaszewski A, Depiesse F, et al. Effects of Ramadan fasting on physical performance and metabolic, hormonal, and inflammatory parameters in middle-distance runners. Appl Physiol Nutr Metab 2009;34:587–94. https://doi.org/10.1139/H09-014.

Farooq A, Herrera CP, Almudahka F, Mansour R. A Prospective Study of the Physiological and Neurobehavioral Effects of Ramadan Fasting in Preteen and Teenage Boys. J Acad Nutr Diet 2015;115:889–97. https://doi.org/10.1016/j.jand.2015.02.012.

Graja A, Ghattassi K, Boudhina N, Bouzid MA, Chahed H, Ferchichi S, et al. Effect of Ramadan intermittent fasting on cognitive, physical and biochemical responses to strenuous short-term exercises in elite young female handball players. Physiol Behav 2021;229:113241. https://doi.org/10.1016/j.physbeh.2020.113241.

Habib AG, Shepherd JC, Eng MKL, Babashani M, Jumare J, Yakubu U, et al. Adherence to anti retroviral therapy (ART) during Muslim Ramadan fasting. AIDS Behav 2009;13:42–5. https://doi.org/10.1007/s10461-008-9412-2.

Haouari-Oukerro F, Ben-Attia M, Kaâbachi N, Haouari M. Ramadan fasting influences on food intake consumption, sleep schedule, body weight and some plasma parameters in healthy fasting volunteers. African Journal of Biotechnology 2013;12.

Herrera CP. Total sleep time in Muslim football players is reduced during Ramadan: a pilot study on the standardized assessment of subjective sleep-wake patterns in athletes. J Sports Sci 2012;30 Suppl 1:S85-91. https://doi.org/10.1080/02640414.2012.676666.

Hsouna H, Abdessalem R, Boukhris O, Trabelsi K, Chtourou L, Tahri N, et al. Short-term maximal performance, alertness, dietary intake, sleep pattern and mood states of physically active young men before, during and after Ramadan observance. PLoS One 2019;14:e0217851. https://doi.org/10.1371/journal.pone.0217851.

Hsouna H, Boukhris O, Trabelsi K, Abdessalem R, Ammar A, Irandoust K, et al. Effects of 25-min nap opportunity during ramadan observance on the 5-m shuttle run performance and the perception of fatigue in physically active men. International Journal of Environmental Research and Public Health 2020;17. https://doi.org/10.3390/ijerph17093135.

Hsouna H, Boukhris O, Trabelsi K, Abdessalem R, Ammar A, Glenn JM, et al. A thirty-five-minute nap improves performance and attention in the 5-m shuttle run test during and outside Ramadan observance. Sports 2020;8:98.

Kadri N, Tilane A, El Batal M, Taltit Y, Tahiri SM, Moussaoui D. Irritability during the month of Ramadan. Psychosom Med 2000;62:280–5. https://doi.org/10.1097/00006842-200003000-00021.

Karli U, Guvenc A, Aslan A, Hazir T, Acikada C. Influence of Ramadan Fasting on Anaerobic Performance and Recovery Following Short time High Intensity Exercise. J Sports Sci Med 2007;6:490–7.

Khalfallah T, Chaari N, Henchi MA, Abdallah B, Ben Chikh R, Saafi MA, et al. Évaluation de l’impact du jeûne du mois du Ramadan sur la charge physique de travail. Archives Des Maladies Professionnelles et de l’Environnement 2004;65:564–70. https://doi.org/10.1016/S1775-8785(04)93519-9.

Laraqui S, Manar N, Laraqui O, Caubet A, Verger C, Laraqui CH. Influence du ramadan vécu sur la vigilance au travail chez les professionnels de santé au Maroc. Archives Des Maladies Professionnelles et de l’Environnement 2012;73:743–9. https://doi.org/10.1016/j.admp.2012.05.002.

Lee JY, San San Tan C, Lee SWH. Ramadan fasting alters sleep behavior in type 2 diabetes patients. Journal of Diabetes 2019;11:93–4.

Leiper JB, Junge A, Maughan RJ, Zerguini Y, Dvorak J. Alteration of subjective feelings in football players undertaking their usual training and match schedule during the Ramadan fast. J Sports Sci 2008;26 Suppl 3:S55-69. <https://doi.org/10.1080/02640410802538176>.

Lessan N, Saadane I, Alkaf B, Hambly C, Buckley AJ, Finer N, et al. The effects of Ramadan fasting on activity and energy expenditure. Am J Clin Nutr 2018;107:54–61. https://doi.org/10.1093/ajcn/nqx016.

Lipert A, Kozłowski R, Rasmus P, Marczak M, Timler M, Timler D, et al. Sleep Quality and Performance in Professional Athletes Fasting during the Month of Ramadan. Int J Environ Res Public Health 2021;18. https://doi.org/10.3390/ijerph18136890.

Magdy R, Kishk NA, Abokrysha NT, Ramzy GM, Rizk HI, Hussein M. Predictors of successful Ramadan fasting in Muslim patients with epilepsy: A prospective study. Seizure 2020;80:67–70. https://doi.org/10.1016/j.seizure.2020.04.012.

Margolis SA, Reed RL. Effect of religious practices of Ramadan on sleep and perceived sleepiness of medical students. Teach Learn Med 2004;16:145–9. https://doi.org/10.1207/s15328015tlm1602_5.

Masood SN, Saeed S, Lakho N, Masood Y, Ahmedani MY, Shera AS. Pre-Ramadan health seeking behavior, fasting trends, eating pattern and sleep cycle in pregnant women at a tertiary care institution of Pakistan. Pakistan Journal of Medical Sciences 2018;34:1326–31. <https://doi.org/10.12669/pjms.346.15883>.

Meckel Y, Ismaeel A, Eliakim A. The effect of the Ramadan fast on physical performance and dietary habits in adolescent soccer players. Eur J Appl Physiol 2008;102:651–7. <https://doi.org/10.1007/s00421-007-0633-2>.

Nassar M, Ahmed TM, AbdAllah NH, El Sayed El Hadidy K, Sheir RE-S. The impact of structured diabetes education on glycemic control during Ramadan fasting in diabetic patients in Beni Suef, Egypt. Diabetes Metab Syndr 2021;15:102249. https://doi.org/10.1016/j.dsx.2021.102249.

Nugraha B, Ghashang SK, Hamdan I, Gutenbrunner C. Effect of Ramadan fasting on fatigue, mood, sleepiness, and health-related quality of life of healthy young men in summer time in Germany: A prospective controlled study. Appetite 2017;111:38–45. <https://doi.org/10.1016/j.appet.2016.12.030>.

Roky R, Chapotot F, Hakkou F, Benchekroun MT, Buguet A. Sleep during Ramadan intermittent fasting. J Sleep Res 2001;10:319–27. https://doi.org/10.1046/j.1365-2869.2001.00269.x.

Roky R, Chapotot F, Benchekroun MT, Benaji B, Hakkou F, Elkhalifi H, et al. Daytime sleepiness during Ramadan intermittent fasting: polysomnographic and quantitative waking EEG study. J Sleep Res 2003;12:95–101. https://doi.org/10.1046/j.1365-2869.2003.00341.x.

Romdhani M, Souissi N, Chaabouni Y, Mahdouani K, Driss T, Chamari K, et al. Improved Physical Performance and Decreased Muscular and Oxidative Damage With Postlunch Napping After Partial Sleep Deprivation in Athletes. Int J Sports Physiol Perform 2020;15:874–83. <https://doi.org/10.1123/ijspp.2019-0308>.

Rosmelia, Setyopranoto I, Hadi H, Wirohadidjojo YW. The effect of ramadan fasting on sebum production: Association with nutritional intakes and sleeping pattern. Bangladesh Journal of Medical Science 2019;18:546–51. https://doi.org/10.3329/bjms.v18i3.41624.

Saddoud A, Khacharem A, H’Mida C, Trabelsi K, Boukhris O, Ammar A, et al. Ramadan Observance Is Associated with Impaired Kung-Fu-Specific Decision-Making Skills. Int J Environ Res Public Health 2021;18. https://doi.org/10.3390/ijerph18147340.

Tian H-H, Aziz A-R, Png W, Wahid MF, Yeo D, Png A-LC. Effects of fasting during Ramadan month on cognitive function in Muslim athletes. Asian Journal of Sports Medicine 2011;2:145.

Trabelsi K, Masmoudi L, Ammar A, Boukhris O, Khacharem A, Jemal M, et al. The effects of Ramadan intermittent fasting on sleep-wake behaviour and daytime sleepiness in team sport referees. J Sports Sci 2021;39:2411–7. https://doi.org/10.1080/02640414.2021.1935672.

Waterhouse J, Alabed H, Edwards B, Reilly T. Changes in sleep, mood and subjective and objective responses to physical performance during the daytime in Ramadan. Biological Rhythm Research 2009;40:367–83. https://doi.org/10.1080/09291010902863438.

Wilson D, Drust B, Reilly T. Is diurnal lifestyle altered during Ramadan in professional Muslim athletes? Biological Rhythm Research 2009;40:385–97. https://doi.org/10.1080/09291010903015996.

Zerguini Y, Kirkendall D, Junge A, Dvorak J. Impact of Ramadan on physical performance in professional soccer players. Br J Sports Med 2007;41:398–400. <https://doi.org/10.1136/bjsm.2006.032037>.

- ***Intermittent fasting***

Bains G, Moh M, Lohman E, Daher N, Silver S, Zamora F, et al. Four weeks of acute intermittent fasting enhances body composition and decreases stress levels in healthy individuals: a pilot study. The FASEB Journal 2020;34:1–1.

Cienfuegos S, Gabel K, Kalam F, Ezpeleta M, Pavlou V, Lin S, et al. The effect of 4-h versus 6-h time restricted feeding on sleep quality, duration, insomnia severity and obstructive sleep apnea in adults with obesity. Nutr Health 2022;28:5–11. https://doi.org/10.1177/02601060211002347.

Gabel K, Hoddy KK, Burgess HJ, Varady KA. Effect of 8-h time-restricted feeding on sleep quality and duration in adults with obesity. Applied Physiology, Nutrition, and Metabolism 2019;44:903–6.

Hutchison AT, Regmi P, Manoogian EN, Fleischer JG, Wittert GA, Panda S, et al. Time‐restricted feeding improves glucose tolerance in men at risk for type 2 diabetes: a randomized crossover trial. Obesity 2019;27:724–32.

Kalam F, Gabel K, Cienfuegos S, Ezpeleta M, Wiseman E, Varady KA. Alternate Day Fasting Combined with a Low Carbohydrate Diet: Effect on Sleep Quality, Duration, Insomnia Severity and Risk of Obstructive Sleep Apnea in Adults with Obesity. Nutrients 2021;13. <https://doi.org/10.3390/nu13010211>.

Kesztyüs D, Fuchs M, Cermak P, Kesztyüs T. Associations of time-restricted eating with health-related quality of life and sleep in adults: a secondary analysis of two pre-post pilot studies. BMC Nutr 2020;6:76. <https://doi.org/10.1186/s40795-020-00402-2>.

Kim H, Jang BJ, Jung AR, Kim J, Ju HJ, Kim YI. The Impact of Time-Restricted Diet on Sleep and Metabolism in Obese Volunteers. Medicina (Kaunas) 2020;56. <https://doi.org/10.3390/medicina56100540>.

Lopes T do VC, Borba ME, Lopes R do VC, Fisberg RM, Lemos Paim S, Vasconcelos Teodoro V, et al. Eating late negatively affects sleep pattern and apnea severity in individuals with sleep apnea. Journal of Clinical Sleep Medicine 2019;15:383–92.

Lowe DA, Wu N, Rohdin-Bibby L, Moore AH, Kelly N, Liu YE, et al. Effects of time-restricted eating on weight loss and other metabolic parameters in women and men with overweight and obesity: the TREAT randomized clinical trial. JAMA Internal Medicine 2020;180:1491–9.

Michalsen A, Schlegel F, Rodenbeck A, Lüdtke R, Huether G, Teschler H, et al. Effects of short-term modified fasting on sleep patterns and daytime vigilance in non-obese subjects: results of a pilot study. Annals of Nutrition and Metabolism 2003;47:194–200.

Park S-J, Yang J-W, Song Y-J. The Effect of Four Weeks Dietary Intervention with 8-Hour Time-Restricted Eating on Body Composition and Cardiometabolic Risk Factors in Young Adults. Nutrients 2021;13. https://doi.org/10.3390/nu13072164.

Parr EB, Devlin BL, Lim KHC, Moresi LNZ, Geils C, Brennan L, et al. Time-restricted eating as a nutrition strategy for individuals with type 2 diabetes: A feasibility study. Nutrients 2020;12:1–22. https://doi.org/10.3390/nu12113228.

Simon SL, Blankenship J, Manoogian EN, Panda S, Mashek DG, Chow LS. The impact of a self-selected time restricted eating intervention on eating patterns, sleep, and late-night eating in individuals with obesity. Frontiers in Nutrition 2022;9:1007824.

Teong XT, Hutchison AT, Liu B, Wittert GA, Lange K, Banks S, et al. Eight weeks of intermittent fasting versus calorie restriction does not alter eating behaviors, mood, sleep quality, quality of life and cognitive performance in women with overweight. Nutr Res 2021;92:32–9. https://doi.org/10.1016/j.nutres.2021.06.006.

Wilkinson MJ, Manoogian ENC, Zadourian A, Lo H, Fakhouri S, Shoghi A, et al. Ten-Hour Time-Restricted Eating Reduces Weight, Blood Pressure, and Atherogenic Lipids in Patients with Metabolic Syndrome. Cell Metab 2020;31:92-104.e5. https://doi.org/10.1016/j.cmet.2019.11.004.

Xie Z, Sun Y, Ye Y, Hu D, Zhang H, He Z, et al. Randomized controlled trial for time-restricted eating in healthy volunteers without obesity. Nature Communications 2022;13:1003.

- ***Day-to-day meal patterns***

Beigrezaei S, Mazidi M, Davies IG, Salehi-Abargouei A, Ghayour-Mobarhan M, Khayyatzadeh SS. The association between dietary behaviors and insomnia among adolescent girls in Iran. Sleep Health 2022;8:195–9.

Faris ME, Vitiello MV, Abdelrahim DN, Cheikh Ismail L, Jahrami HA, Khaleel S, et al. Eating habits are associated with subjective sleep quality outcomes among university students: findings of a cross-sectional study. Sleep and Breathing 2021:1–12.

Tahara Y, Makino S, Suiko T, Nagamori Y, Iwai T, Aono M, et al. Association between irregular meal timing and the mental health of Japanese workers. Nutrients 2021;13:2775.

**Meal frequency**

Beigrezaei S, Mazidi M, Davies IG, Salehi-Abargouei A, Ghayour-Mobarhan M, Khayyatzadeh SS. The association between dietary behaviors and insomnia among adolescent girls in Iran. Sleep Health 2022;8:195–9.

Fujii H, Miyamoto M, Miyamoto T, Hirata K, Muto T. Relationship between daily meal frequency and subjective sleep quality or daytime sleepiness in Japanese medical students 2009.

Reid KJ, Baron KG, Zee PC. Meal timing influences daily caloric intake in healthy adults. Nutrition Research 2014;34:930–5.
