# Supplementary material for: Chrono-nutrition and sleep: lessons from the temporal feature of eating patterns in human studies - A systematic scoping review
Source: Sleep Med Rev. Author manuscript; Available in PMC 2025 Aug 1. (PMC12090848; doi:10.1016/j.smrv.2024.101953)
Supplement: Supportive file A [file NIHMS2076074-supplement-Supportive_file_A.docx]

**Chrono-nutrition and sleep - lessons from the temporal feature of eating patterns in human studies: A systematic scoping review**

**Authors**

Oussama Saidi ^1*^, Emmanuelle Rochette^1,2,3^, Lou Dambel ^1^, Marie-Pierre St-Onge ^4^, Pascale Duché ^1^

**Affiliations**

1. JAP2S laboratory, Toulon University, F-83041 Toulon, France.
2. Department of Pediatrics, Clermont-Ferrand University Hospital, F-63000 Clermont-Ferrand, France
3. Clermont Auvergne University, INSERM, CIC 1405, CRECHE unit, F-63000 Clermont-Ferrand, France
4. Center of Excellence for Sleep and Circadian Research and Division of General Medicine, Department of Medicine, Columbia, University Irving Medical Center, New York, NY, USA 10032.

**OCRID IDs**

Oussama Saidi: 0000-0003-3005-8716

Emmanuelle Rochette: 0000-0001-5180-9916

Marie-Pierre St-Onge : 0000-0003-1354-1749

Pascale Duché: 0000-0001-7206-6429

***Corresponding author**

Dr. Oussama SAIDI, IAPS Laboratory, Toulon University, F-83041 Toulon, France

E-mail: oussama.saidi@univ-tln.fr

Tel: +33 6 58 48 72 88

**Financial disclosure:**

Marie-Pierre St-Onge is funded in part by the National Institutes of Health grants R01 DK128154, R01 HL142648 and R35 HL155670

Supporting File A (online supplement)

**Tables**

- **Table S1.** Search strategy for each database
- **Table S2.** Characteristics and main findings of studies exploring the effect of meal timing on sleep
- **Table S3.** Characteristics and main findings of studies exploring the effect of irregular meal pattern on sleep
- **Table S4.** Characteristics and main findings of studies exploring the effect of meal frequency on sleep

**Figures**

- **Figure S1.** Meal timing dimension detailed tabular representation of each study sleep outcomes
- **Figure S2.** Diurnal fasting and intermittent fasting sub-dimensions detailed tabular representation of each study sleep outcomes

**Table S1.** Search strategy for each database

| **PubMed Session Results** | | Number of records |
| --- | --- | --- |
| **Chrono-nutrition** | ("chrononutrition"[All Fields] AND ("sleep"[MeSH Terms] OR "sleep"[All Fields] OR "sleeping"[All Fields] OR "sleeps"[All Fields] OR "sleep s"[All Fields])) Filters: Humans, English | 69 |
| **Frequency** | (("meal"[Title] OR "eating"[Title] OR "feeding"[Title] OR “breakfast”[Title]) AND ("timing"[Title] OR “late”[Title] OR “early”[Title]) AND ("sleep"[MeSH Terms] OR "sleep"[All Fields] OR "sleeping"[All Fields] OR "sleeps"[All Fields] OR "sleep s"[All Fields])) Filters: Humans, English | 38 |
| **Timing** | (("meal"[Title] OR "eating"[Title] OR "feeding"[Title] OR “breakfast”[Title]) AND ("timing"[Title] OR “late”[Title] OR “early”[Title]) AND ("sleep"[MeSH Terms] OR "sleep"[All Fields] OR "sleeping"[All Fields] OR "sleeps"[All Fields] OR "sleep s"[All Fields])) Filters: Humans, English | 78 |
| **Regularity** | (("meal"[Title] OR "eating"[Title] OR "feeding"[Title] OR “breakfast”[Title]) AND ("regularity"[Title] OR "stability"[Title] OR "irregularity"[Title] OR "pattern"[Title] OR "consistency"[Title] OR "inconsistency"[Title]) AND ("sleep"[MeSH Terms] OR "sleep"[All Fields] OR "sleeping"[All Fields] OR "sleeps"[All Fields] OR "sleep s"[All Fields])) Filters: Humans, English | 14 |
| **Irregular meal pattern** | - Ramadan: (“Ramadan"[Title] AND ("sleep"[MeSH Terms] OR "sleep"[Title/Abstract] OR "sleeping"[Title/Abstract] OR "sleeps"[Title/Abstract] OR "sleep s"[Title/Abstract])) Filters: Humans, English - Other religious fasts: ((“religious”[Title/Abstract] OR “religion”[Title/Abstract]) AND (“eating”[Title] OR “fasting”[Title] OR “meal”[Title] OR “diet”[Title]) AND ("sleep"[MeSH Terms] OR "sleep"[Title/Abstract] OR "sleeping"[Title/Abstract] OR "sleeps"[Title/Abstract] OR "sleep s"[Title/Abstract])) Filters: Humans, English - Intermittent fasting: ((“intermittent” [Title] OR “restricted”[Title] OR “modified”[Title] OR “reduced”[Title] OR “limited”[Title] OR “controlled”[Title] OR “delayed”[Title] OR “intermittent”[Title]) AND (“eating”[Title] OR “fasting”[Title] OR “meal”[Title] OR “diet”[Title]) AND ("sleep"[MeSH Terms] OR "sleep"[Title/Abstract] OR "sleeping"[Title/Abstract] OR "sleeps"[Title/Abstract] OR "sleep s"[Title/Abstract])) Filters: Humans, English - Alternate day fasting: ((“alternate-day”[Title] OR “alternate day”[Title]) AND (“eating”[Title] OR “fasting”[Title] OR “meal”[Title] OR “diet”[Title]) AND ("sleep"[MeSH Terms] OR "sleep"[Title/Abstract] OR "sleeping"[Title/Abstract] OR "sleeps"[Title/Abstract] OR "sleep s"[Title/Abstract])) Filters: Humans, English - Time restricted eating/feeding: (“timed”[Title] OR “time”[Title] OR “time-restricted”[Title] OR “16:8”[Title] OR “Warrior”[Title]) AND (“eating”[Title] OR “fasting”[Title] OR “meal”[Title] OR “diet”[Title]) AND ("sleep"[MeSH Terms] OR "sleep"[Title/Abstract] OR "sleeping"[Title/Abstract] OR "sleeps"[Title/Abstract] OR "sleep s"[Title/Abstract])) Filters: Humans, English - Modified fasting regimens/intermittent energy restriction: (("intermittent energy restriction"[Title/Abstract] OR "modified fasting regimen"[Title/Abstract] OR "5:2 diet"[Title/Abstract]) AND ("sleep"[MeSH Terms] OR "sleep"[Title/Abstract] OR "sleeping"[Title/Abstract] OR "sleeps"[Title/Abstract] OR "sleep s"[Title/Abstract])) Filters: Humans, English | 242 |
| **ScienceDirect (Elsevier) Session Results** | | |
| **Chrono-nutrition** | Title, abstract, keywords: chrononutrition  And all text: sleep  Filters: Review articles and research articles | 12 |
| **Frequency** | Title: (meal OR eating OR feeding OR breakfast) AND (occasion OR number OR  frequency OR skipping) AND All text: sleep  Filters : Review articles and research articles | 36 |
| **Timing** | Title: (meal OR eating OR feeding OR breakfast) AND (timing OR late OR early) AND All text : sleep  Filters: Review articles and research articles | 81 |
| **Regularity** | Title: (meal OR eating OR feeding OR breakfast) AND (regularity OR stability OR irregularity OR  Pattern OR consistency) AND All text: sleep  Filters: Review articles and research articles | 95 |
| **Irregular meal pattern** | - Ramadan: Title: ramadan AND All text: sleep Filters: Review articles and research articles - Other religious fasting: Title (religious OR religion) AND (eating OR fasting OR meal OR diet) AND All text: sleep Filters: Review articles and research articles - Intermittent fasting: Title: (intermittent OR restricted OR limited OR controlled OR delayed) AND (eating OR fasting OR meal) AND All text: sleep - Alternate day fasting: Title (alternate day OR alternate-day) AND (eating OR fasting OR meal OR diet) AND All text: sleep Filters: Review articles and research articles - Time restricted eating/feeding: Title (timed OR time-restricted OR time OR 16:8 OR warrior) AND (eating OR fasting OR meal OR diet) AND All text: sleep Filters: Review articles and research articles - Modified fasting regimens/intermittent energy restriction: Title, abstract (intermittent energy restriction OR modified fasting regimen OR 5:2 diet) AND All text: sleep Filters: Review articles and research articles | 342 |
| **Scopus Session Results** | | |
| **Chrono-nutrition** | TITLE-ABS-KEY (chrononutrition) AND (LIMIT-TO (DOCTYPE, "ar") OR LIMITTO (DOCTYPE, "re")) AND (LIMIT-TO (EXACTKEYWORD, "Human")) AND ( LIMITTO (LANGUAGE, "English")) | 76 |
| **Frequency** | TITLE (meal OR eating OR feeding OR breakfast) AND TITLE (occasion OR frequency OR consistency OR pattern) AND TITLE-ABS-KEY (sleep) AND (LIMIT-TO ( DOCTYPE, "ar") OR LIMIT-TO (DOCTYPE, "re")) AND (LIMIT-TO (LANGUAGE, "English")) AND (LIMIT-TO ( EXACTKEYWORD, "Human")) | 75 |
| **Timing** | TITLE (meal OR eating OR feeding OR breakfast) AND TITLE (timing OR late OR early) AND TITLE-ABS-KEY (sleep) AND (LIMIT-TO ( DOCTYPE, "ar") OR LIMIT-TO (DOCTYPE, "re")) AND (LIMIT-TO (LANGUAGE, "English")) AND (LIMIT-TO (EXACTKEYWORD, "Human")) | 97 |
| **Regularity** | TITLE (meal OR eating OR feeding OR breakfast) AND TITLE (regularity OR stability OR irregularity OR pattern OR consistency) AND TITLE-ABS-KEY (sleep) AND (LIMIT-TO (DOCTYPE, "ar") OR LIMIT-TO (DOCTYPE, "re")) AND (LIMIT-TO (LANGUAGE, "English" )) AND ( LIMIT-TO ( EXACTKEYWORD , "Human" )) | 60 |
| **Irregular meal pattern** | - Ramadan: TITLE (ramadan) AND TITLE-ABS-KEY (sleep) AND (LIMIT-TO (DOCTYPE, "ar") OR LIMIT-TO (DOCTYPE, "re")) AND (LIMIT-TO (LANGUAGE, "English")) AND (LIMIT-TO (EXACTKEYWORD, "Human")) - Other religious fasting: TITLE (religious OR religion) AND TITLE eating OR fasting OR meal OR diet) AND TITLE-ABS-KEY (sleep) AND (LIMIT-TO (DOCTYPE, "ar") OR LIMIT-TO (DOCTYPE, "re")) AND (LIMIT-TO (LANGUAGE, "English")) AND (LIMIT-TO (EXACTKEYWORD, "Human")) - Intermittent fasting: TITLE (intermittent OR restricted OR reduced OR limited OR controlled OR delayed) AND TITLE (eating OR fasting OR meal OR diet) AND TITLE-ABS-KEY (sleep) AND (LIMIT-TO (DOCTYPE, "ar") OR LIMIT-TO (DOCTYPE, "re")) AND (LIMIT-TO (LANGUAGE, "English")) AND (LIMIT-TO (EXACTKEYWORD, "Human")) - Alternate day fasting: TITLE (alternate day OR alternate-day) AND TITLE (eating OR fasting OR meal OR diet) AND TITLE-ABS-KEY (sleep) AND (LIMIT-TO (DOCTYPE, "ar") OR LIMIT-TO (DOCTYPE, "re")) AND (LIMIT-TO (LANGUAGE, "English")) AND (LIMIT-TO (EXACTKEYWORD, "Human")) - Time restricted eating/fasting: TITLE (timed OR time-restricted OR time OR 16:8 OR warrior) AND TITLE (eating OR fasting OR meal OR diet) AND TITLE-ABS-KEY (sleep) AND (LIMIT-TO (DOCTYPE, "ar") OR LIMIT-TO (DOCTYPE, "re")) AND (LIMIT-TO (LANGUAGE, "English")) AND (LIMIT-TO (EXACTKEYWORD, "Human")) - Modified fasting regimens/intermittent energy restriction: TITLE-ABS-KEY (intermittent energy restriction OR modified fasting regimen OR 5:2 diet) AND TITLE-ABS-KEY (sleep) AND (LIMIT-TO (DOCTYPE, "ar") OR LIMIT-TO (DOCTYPE, "re")) AND (LIMIT-TO (LANGUAGE, "English")) AND (LIMIT-TO (EXACTKEYWORD, "Human")) | 519 |

**Table S2.** Characteristics and main findings of studies exploring the effect of meal timing on sleep

| Study | Design | Sample size | Participants’ characteristics | Intervention / duration | Sleep measurement and outcomes | | Main findings |
| --- | --- | --- | --- | --- | --- | --- | --- |
| ***** Breakfast skipping*** (n=13)** | | | | | | | |
| Azemati et al. (2020) | Cross-sectional | 14274 | Healthy  Age: 7-18 y  50.6% males, 49.4% females  BMI: ND | N/A | ⬠ | Difficulties in getting to sleep | - Breakfast skipping was associated with increased sleep difficulty - Dinner skipping was associated with increased sleep difficulty |
| Beigrezaei et al. (2022) | Cross-sectional | 988 | Females, healthy  Age: 12-18 y  BMI: ND | N/A | ⬠ | Insomnia Severity Index | - Increased rates of insomnia among participants with lowest frequency of eating breakfast |
| Faris et al. (2021) | Cross-sectional | 498 | Healthy students  Age: 18-30 y  185 males, 313 females  BMI: ≤ 24.9 kg/m^2^ (n=310), 25–29.9 kg/m^2^ (n=106), ≥ 30–34.9 kg/m^2^ (n=82) | N/A | ⬠ | PSQI | - Skipping breakfast was correlated with poor sleep quality (r = − 0.111, p = 0.007) |
| Gwin et al. (2018) | Crossover RCT | 13 | Healthy  Age: 20-32 y  6 males, 7 females  BMI <25 kg/m^2^ | High protein breakfast vs. breakfast skipping for 7 days | ⬟ AND ⬠ | Accelerometry   - TST - SE   Pittsburgh Sleep Diary   - Sleep quality - SOL - Number of awakenings - Daytime sleepiness | - Higher TST during no breakfast session compared to breakfast - No differences in sleep efficiency or daytime sleepiness - Better perceived sleep quality and onset during breakfast session |
| Lebacq et al. (2022) | Cross-sectional | 8444 | Healthy  Age: 11-20 y  4129 males, 4315 females  BMI: ND | N/A | ⬠ | Sleep survey   - TST | - Lower sleep duration in breakfast skipper compared to breakfast eaters - Sleep duration was positively associated with daily breakfast consumption after adjustment for morning tiredness (aOR = 1.28 ; 95% CI 1.21–1.35) |
| Lopes et al. (2019) | Cross-sectional | 296 | Mild to severe OSA  Age: 20 to 60 y  211 males, 85 females  BMI: >25 kg/m^2^ | N/A | ⬟ AND ⬠ | PSG   - TST - SE - SOL - WASO - Sleep staging   PSQI  Epworth Sleepiness Scale | - Skipping breakfast was associated with later bedtime and with reduced TST (p<0,05) compared to both early and late breakfast eaters |
| Liu et al. (2022) | Cross-sectional | 5254 | Healthy  Age: 6-17 y  2785 males, 2469 females  BMI: ND | N/A | ⬠ | Sleep survey   - TST | - Higher rate of skipping breakfast was related to short sleep duration (slight to severe) |
| Manmee et al. (2017) | Cross-sectional | 356 | Hospital personnel  Age: 38.7 ± 11.4 y  46 males, 310 females BMI <25 kg/m^2^ | N/A | ⬠ | Sleep survey   - TST - Sleep quality - Sleep hygiene | - Lower sleep quality and delayed bedtime were more prevalent among breakfast skippers compared to eaters |
| Reutrakul et al. (2014) | Cross-sectional | 194 | T2DM  172 breakfast eaters:  Age: 59.5 ± 12.4 y  BMI: 35.1 ± 8.2 kg.m^2^  22 breakfast skipper:  Age: 49.6 ± 14.6 y  59 males, 135 females  BMI: 40.0 ± 7.2 | N/A | ⬠ | Sleep survey  for 1 month   - Bedtime - Wake up time - SOL - TST - Perceived sleep debt | - Delayed sleep in breakfast skippers compared to breakfast eaters (bedtime, wake up time / sleep duration - No difference in perceived sleep debt |
| Tambalis et al. (2019) | Cross-sectional | 177 091 | Healthy  Age: 8-17 y  90 316 males, 86 775 females  BMI: 19.7 ± 3.8 kg.m^2^ | N/A | ⬠ | - TST | - Reduced TST in breakfast skippers compared to breakfast eaters |
| Thivel et al. (2015) | Cross-sectional | 236 | Healthy  Age: 6-10 y  112 males, 224 females  BMI: ND | N/A | ⬠ | 7-days parent-reported   - Bedtime - Wake up time - TST | - Bedtime was significantly earlier in children consuming breakfast everyday ‘8:30 vs 9:00 p.m, p<0.01 |
| Yasuda et al. (2018) | Cross-sectional | 270 | College and graduate school students (3 groups)  152 males, 118 females  Male aged: 22.2 ± 2.3; 21.5 ± 2.3; 20.5 ± 2.0 y  Female aged: 22.2 ± 3.4; 21.0 ± 1.8; 21.2 ± 2.4 y  BMI: < 25 kg.m^2^ | N/A | ⬠ | PSQI   - Bedtime - Wake up time - SOL - TST - Sleep quality | - Significant differences were found for several PSQI outcomes according to breakfast skipping frequency in both males and females (bedtime, wake up time, sleep latency, sleep duration, sleep efficiency and overall sleep quality) - No differences were found for sleep duration |
| Zhou et al. (2022) | Cross-sectional | 1063 | College students  Age: 17-26 y, (Mean age: 19.8 ± 1.3 y)  415 males, 648 females  BMI: ND | N/A | ⬠ | PSQI | - Skipping breakfast more frequently was associated with a lower sleep quality in female (OR: 1.332 (1.031~1.721) |
| ***** Late Eating *** (n=16)** | | | | | | | |
| Chung et al. (2020) | Cross-sectional | 793 | University students  Age: 18-29 y  206 males, 587 females  BMI: ≤ 24.9 kg/m^2^ (n=650), 25–29.9 kg/m^2^ (n=113), ≥ 30–34.9 kg/m^2^ (n=30) | N/A | ⬠ | Online sleep survey | - Consuming food within 3 hours of bedtime was linked with increased WASO (odds ratio = 1.61, 95% CI = 1.15–2.27) - No significant association of consuming food with 3 hours of bedtime with SOL (1.24; 0.89–1.73) or short TST (0.79; 0.49–1.26) |
| Crispim et al. (2011) | Cross-sectional | 52 | Healthy  Age: males 27.2 ± 5.9 y, females 28.8 ± 6.6 y  25 males, 27 females  BMI <25 kg/m^2^ | N/A | ⬟ | PSG   - TST - SE - SOL - WASO - Sleep staging | - Food intake near the sleeping period (dinner and late-night snack) was correlated with increased SOL, and decreased SE |
| Driver et al. (1999) | Crossover RCT | 7 | Males, healthy  Age : 20–24 y  BMI : 23.4 ± 2.6 kg/m^2^ | 4 nights (an adaptation night, followed by either of the two meal conditions or the fast in random order) | ⬟ | PSG   - TST - SE - SOL - WASO - Sleep staging | - No differences in sleep outcomes between all conditions (Fast, control, high energy evening meal) |
| Duan et al. (2021) | Crossover RCT | 20 | Healthy  Age: 26 ± 2.7 y  10 males, 10 females  BMI: 23.2 ± 3.1 kg/m^2^ | Routine dinner (5 h before bedtime) vs. later dinner (1h before bedtime) | ⬟ | PSG   - TST - SE - SOL - WASO - Sleep staging - EEG spectral analysis | - Conventional sleep stages were similar between the 2 sessions - Later dinner caused a 2.5% initial increase in delta power and a reciprocal 2.7% decrease in combined alpha and beta power (p<0,001) - These effects diminished as sleep continued with a reversal of these patterns in the latter part of the night |
| Falkenberg et al. (2021) | Prospective | 36 | Males, athletes  Age: 23.5 ± 3.9 y  Body mass: 86.6 ± 8.1 kg | Assessment for 10 consecutive days | ⬟ AND ⬠ | Accelerometry   - TST - SOL - SE - WASO   Sleep survey | - Evening protein intake was associated with shortened SOL - Evening sugar intake was associated with shorter TST - Longer period between the evening meal consumption and bedtime was associated with a shorter TST |
| Faris et al. (2021) | Cross-sectional | 498 | Students  Age: 18-30  185 males, 313 females  BMI: ≤ 24.9 kg/m^2^ (n=310), 25–29.9 kg/m^2^ (n=106), ≥ 30–34.9 kg/m^2^ (n=82) | N/A | ⬠ | PSQI | - Late-night snacks (r = − 0.109, p = 0.007) intake wad correlated with poor sleep quality |
| Garaulet et al. (2013) | NRS | 420 | Early-eaters (51%) and late-eaters (49%)  Age: 42 ± 11 y  212 males; 208 females  BMI: 31.4 ± 5.4 kg/m^2^ | 20 weeks of weight loss intervention  Comparison of late-eaters and early-eaters | ⬠ | Sleep survey  TST (week and weekend) | - No differences in sleep duration between early-eaters and late-eaters at intervention endpoint |
| Hermenegildo-López et al (2021) | Prospective  (only baseline outcomes were reported) | 607 | Free from MetS at baseline  Age ≥ 60 y  295 males, 312 females  BMI: 27.5 ± 3.4 kg/m^2^ | N/A | ⬠ | TST | - No differences in sleep duration according to quartile of % EI eaten at breakfast, lunch and dinner |
| Lehmann et al. (2022) | Cross-over | 12 | Males, athletes  Age: 15.8 ± 0.7 y  BMI: 25.8 ± 4.1 kg/m^2^  FM: 16.8 ± 6 % | Five consecutive days in each of two conditions: routine dinner (3.5 h before bedtime) and late dinner (LD, 1.5 h before bedtime) | ⬟ | PSG   - TST - SE - SOL - WASO - Sleep staging   Sleep survey | - Increase in TST and SE in late dinner compared to routine dinner - No significant differences in sleep architecture and SOL |
| Lopes et al. (2019) | Cross-sectional | 296 | Mild to severe OSA  Age: 20 to 60 y  211 males, 85 females  BMI: > 25 kg/m² | N/A | ⬟ AND ⬠ | PSG   - TST - SE - SOL - WASO - Sleep staging   PSQI  Epworth Sleepiness Scale | - PSG: habitual late meal intake was associated with worse measured sleep parameters (↑ SOL, ↑ WASO, ↑ N1 absolute time, ↑ AHI) - Eating late was associated with poor sleep quality (PSQI) - Eating duration was associated with ↑ ESS |
| Martínez-Lozano et al. (2020) | Cross-sectional | 397 | Healthy, late dinner eaters (n=197) and early dinner eaters (n=200)  Age: 8 to 12 y  196 males, 201 females  BMI (range): 19.4 kg/m^2^ (11.6–35.1) | N/A | ⬟ AND ⬠ | Accelerometry   - TST   Sleep diary   - Bedtime - Wake up time   Sleep survey   - TST | - No differences between LDE and EDE groups in objectively measured TST - Time in bed was delayed in LDE compared to EDE |
| Orr et al. (1998) | Crossover RCT | 20 | Symptomatic reflux  Age: 25 to 68 y  12 males, 8 females  BMI: ND | Early dinner (19:00) vs. Late dinner (21:00), under fixed time in bed condition | ⬟ | PSG   - TST - SE - SOL - WASO - Sleep staging | - ↓ REM absolute time following late dinner - No differences between the two conditions for all the remaining sleep parameters |
| Reid et al. (2014) | Prospective | 59 | Healthy  Age: 31.7 ± 11.8 y  29 males, 30 females  BMI: 24.1 ± 4.2 kg/m² | Seven days assessment | ⬟ AND ⬠ | Accelerometry   - TST   Sleep survey | - TST was correlated with last meal, and window between last-meal and sleep onset - Eating late and eating closer to sleep onset was associated with a decrease in TST |
| Saidi et al. (2021) | Crossover RCT | 28 | Males with BMI ≥ 90th centile and healthy age matched controls  Age: 14 ± 0.9 y  BMI: 33.1 ± 7.1 kg/m², 20.71 ± 1.58 kg/m² | Eucaloric vs. ad libitum diet | ⬟ | PSG   - TST - SE - SOL - WASO - Sleep staging | - Increased % EI during dinner was associated with decreased SE (p < 0.01), N3 stage (p < 0.01), and increased stage 2 sleep (p < 0.05) |
| Soreca et al. (2016) | Cross-sectional | 114 | Bipolar disorder  Age: 41.5 ± 9.5 y  47 males, 67 females  BMI: 34.9 ± 6.9 kg/m² | N/A | ⬟ AND ⬠ | Accelerometry   - TST   PSQI | - Mean dinner time was not significantly correlated to TST |
| Uçar et al. (2021) | Crossover RCT | 16 | Males, healthy  Age: 20 to 26 y  BMI: 23.2 kg/m^2^ (20.9-26.7) | No dinner vs. Easily-digestible meal vs. slowly-digestible meal (protein+ fat-rich, i.e. kebab) in the last hour before going to sleep | ⬠ | Sleep diary  PSQI  KSS | - No differences regarding sleep awakening and TST - Higher subjective sleep disturbances score in the late slowly-digestible meal condition |
| ***** Shift in meal schedules ***(n=6)** | | | | | | | |
| Allison et al. (2021) | Crossover | 12 | Healthy  Age: 26.3 ± 3.4 y  7 males, 5 females  BMI: 21.9 ± 1.7 kg/m² | Eight-week conditions, twice: daytime (intake limited to 08h–19h) and delayed (intake limited to 12h–23h) with two-week washout | ⬟ | Accelerometry   - TST - SOL - SE | - Sleep-wake cycles did not differ between the two conditions |
| Bazzani et al. (2022) | Cross-sectional | 1298 | Healthy  Age: 39.17±14.45 y  495 males, 803 females  BMI: ≤ 24.9 kg/m^2^ (n=920), 25–29.9 kg/m^2^ (n=306), ≥ 30–34.9 kg/m^2^ (n=71) | N/A | ⬠ | PSQI | - Consuming first and last eating event was found to be preferred to be later in bad sleepers compared to good sleepers |
| Loo et al. (2022) | Cross-sectional | 90 | Pregnant women in the second trimester  Age: ≥ 18 y  BMI: 23.1 ± 4.0 kg/m^2^ | One shot data from a cohort study on maternal night-eating patterns | ⬠ | PSQI | - Women with poor sleep, depression, anxiety, and stress symptoms higher odds of meal skipping (OR 1.99; 95% CI 1.13, 3.53) and meal delaying (2.50; 1.31, 4.79) |
| Pizinger et al. (2018) | Cross-over | 6 | Age: 25.1 ± 3.9 y  4 males, 2 females  BMI: 29.2 ± 2.7 kg/m² | 4 conditions randomized crossover inpatient study differing in sleep times: normal or late and in meal times: normal or late | ⬟ | Accelerometry   - TST - SOL - SE - WASO   PSG   - TST - SE - SOL - WASO - Sleep staging | - No difference in TST, SE, SOL, number of awakenings, and WASO or sleep stage assessed by PSG |
| Ruddick-Collins et al. (2022) | Cross-over | 30 | Age: 50.9 ± 2.1 y  16 males, 14 females  BMI: 32.5 ± 0.7 kg/m² | 4-week calorie-restricted but isoenergetic weight loss diets, with morning loaded or evening loaded calories | ⬟ AND ⬠ | Sleep survey   - TST - SOL, - Sleep quality   Accelerometry   - TST | - No changes in TST assessed by accelerometry or self-reported |
| Wehrens et al. (2017) | Cross-over | 10 | Males, healthy  Age: 18-30 y  BMI: 23.1 ± 0.8 kg/m² | 13 day laboratory protocol, early meals for 6 days then late meals for 6 days | ⬟ AND ⬠ | Accelerometry  KSS | - No significant effect on sleepiness - Meal timing did not alter sleep parameters before circadian rhythm measurement |
| AHI: apnea hypopnea index; BMI: body mass index; CI: confidence interval; EDE: early dinner eaters; EEG: electroencephalography; EI: energy intake; ESS: Epworth Sleepiness Scale; GRT: group randomized trials; KSS: Karolinska Sleepiness Scale; LDE: late dinner eaters; MetS: metabolic syndrome; N/A: not applicable; NRS: non randomized studies; OR: odds ratio; OSA: obstructive sleep apnea; PSG: polysomnography; PSQI: Pittsburgh Sleep Quality Index; RCT: randomized controlled trials; RD: routine dinner; REM: rapid-eye movement sleep; SE: sleep efficiency; SOL: sleep onset latency; T2DM: type 2 diabetes mellitus; TST: total sleep time; WASO: wake after sleep onset; ↓: decrease; ↑: increase; ⬟: objective measures; ⬠: self-reported measures | | | | | | | |

**Table S3.** Characteristics and main findings of studies exploring the effect of irregular meal pattern on sleep

| Study | Design | Sample size | Participants’ characteristics | Intervention / duration | Sleep measurement and outcomes | | Main findings |
| --- | --- | --- | --- | --- | --- | --- | --- |
| ***** Diurnal fasting*** (n=65)** | | | | | | | |
| Abdul Razzak et al. (2019) | GRT | 39 | Males, healthy  Age: 18-23 y  BMI: ND | Fasting at fixed times of the day during the month of Ramadan | ⬠ | Self-reported | - Fasting status had no effect on TST |
| Akbari et al. (2022) | cross-sectional | 510 | Healthy fasting state  Age: 31 ± 12 y  179 males, 331 females  BMI: ND | Fasting at fixed times of the day during the month of Ramadan | ⬠ | PSQI | - ↓ sleep-quality of the active participants who fasted |
| Al-Rawi et al. (2020) | prospective | 57 | Age: 38.4 ± 11.2 y  40 males, 17 females  BMI > 25 kg/m^2^ | Fasting at fixed times of the day (11:00-1:00) during the month of Ramadan | ⬠ | Sleep diary  TST | - ↓ TST during the month of Ramadan compared to BR (p<0.001) |
| Alghamdi et al. (2020) | prospective | 36 | T2DM  Age: 49.50 ± 11.91 y  20 males, 16 females  BMI: 32.89 ± 5.47 kg/m^2^ | Fasting at fixed times of the day during the month of Ramadan | ⬟ AND ⬠ | Accelerometry   - TST - Total time awake   Sleep survey   - sleep patterns - TST | - ↓ TST (6.03 h vs 7.02 h; p <0.001) |
| Almeneessier et al. (2017) | prospective | 8 | Males, healthy  Age: 26.6 ± 4.9 y  BMI: 23.7 ± 3.5 kg/m^2^ | BR: dinner at 20:00, breakfast at 07:15, and lunch at 12:00 mid‑day.  During fasting Ramadan: breakfast (at sunset, between 18:30 and 18:55 h), dinner at 21:00 h, and suhur before dawn (between 03:00 and 03:15 h) | ⬟ | PSG   - TST - SE - SOL - WASO - Sleep staging   Accelerometry   - TST | - Delay in bedtime and wake-up time during Ramadan - SE was lower during Ramadan (74.2% ± 7.3%) than BR (79.3% ± 9.6%), p=0.3 |
| Almeneessier et al. (2019) | prospective | 8 | Males, healthy  Age: 24.4 ± 3.7 y  BMI: 23.8 ± 4.1 kg/m^2^ | Fasting at fixed times of the day during the month of Ramadan | ⬟ | PSG   - TST - SE - SOL - WASO - Sleep staging   Accelerometry   - TST - SE | - Bed time and wake time during Ramadan are delayed - No change in TST assessed by accelerometry - ↓ REM sleep percentage during fasting |
| Almeneessier et al. (2019) | prospective | 12 | Males, healthy  Age: 25.1 ± 2.5 y  BMI: 23.4 ± 3.5 kg/m^2^ | Light meal was served at sunset to break the fast, followed by a dinner meal at 21:00 and a pre-dawn meal 30 min before dawn time. Sunset and dawn times ranged from 18:23 and 04:02 | ⬟ | PSG   - TST - SE - SOL - WASO - Sleep staging   Accelerometry   - TST | - No differences in TST, SOL, arousal index, stage shifts, and SE |
| Alzhrani et al. (2022) | prospective | 115 | Healthy  Age: 29.6 ± 6.6 y  19 males, 96 females  BMI: 26.4 ± 6.1 kg/m^2^ | Fasting at fixed times of the day during the month of Ramadan. | ⬠ | Epworth Sleepiness Scale | - ↑ percentage of participants in the normal range of sleepiness (from 0 to 10) during Ramadan - TST did not change |
| Aziz et al. (2010) | prospective | 10 | Males, moderately trained  Age: 27.3 ± 7.2 y  BMI < 25 kg/m^2^ | Fasting at fixed times of the day (05:25 -19:10) during the month of Ramadan | ⬠ | Karolinska Sleepiness Scale  Sleep diary   - TST | - No impact on daytime sleepiness - ↓ TST during Ramadan as compared to before or after Ramadan (4.7 ± 1.5h *vs* 7.7 ± 1.5h, p < 0.01) |
| Aziz et al. (2017a) | prospective | 14 | Males, football players Age: 21.8 ± 2.4 y  BMI < 25 kg/m^2^ | Fasting at fixed times of the day during the month of Ramadan. Controlling for Pre-assessment Dietary Intake | ⬟  AND  ⬠ | Accelerometry   - TST   Epworth Sleepiness Scale | - No significant differences in TST (p=0.87) and daytime sleepiness (p=0.25) |
| Aziz et al. (2017b) | NRS | 13 | Males, trained soccer players  Age: 20.1 ± 0.9 y  BMI < 25 kg/m^2^ | Fasting at fixed times of the day during the month of Ramadan | ⬠ | Epworth Sleepiness Scale  Sleep survey | - No significant effect on TST - During RA: fasted players spent more time napping in the afternoon (p = 0.02) - No difference in ESS (p = 0.97) |
| Aziz et al. (2020) | NRS | 10 | Males, national-level badminton players  Age: 22.8 ± 3.4 y  BMI < 25 kg/m^2^ | Fasting at fixed times of the day during the month of Ramadan | ⬠ | TST  Brunel Mood State  Karolinska Sleepiness Scale | - ↑ BRUMS levels of subjective fatigue in Ramadan group compared with control (P < 0.001) - Overnight sleep duration (p = 0.49), daytime nap duration (p = 0.10) and KSS scores (p = 0.24) were similar between groups |
| BaHammam et al. (2003) | prospective | 56 | Healthy  Age: 22.6 ± 1.3 y  31males, 25 females  BMI: 23.4 ± 4.0 kg/m^2^ | Fasting at fixed times of the day during the month of Ramadan | ⬠ | Epworth Sleepiness Scale | - During Ramadan: Bedtime and wake time delayed - ↓ TST (p > 0.05) - ↑ proportion of students who nap from X to 73% - No difference in TST + naps between BR (7.25 ± 2 h) and Ramadan (RA1 7.22 ± 1.5 h; RA2 6.85 ± 1.5 h; and RA3 6.8 ± 1.9 h) - ↑ ESS scores |
| BaHammam et al. (2004) | prospective | 8 | Males, healthy  Age: 31.8 ± 2 y  BMI: 25.0 ± 2.2 kg/m^2^ | fasting at fixed times of the day during the month of Ramadan | ⬟  AND  ⬠ | PSG   - TST - SE - SOL - WASO - Sleep staging   Multiple sleep latency test  Epworth Sleepiness Scale | - ESS: ↓ TST 6.8 ± 1.1h at BR vs 4.7 ± 1.7h at RA1 and RA3 (p<0.05) - PSG: no change in TST, ↓ SOL at RA3 compared to BR (18.5 ± 10.0 min vs 35.0 ± 12.0; p = 0.05) - No significant difference in REM latency at RA1 (106 ± 42 min) and RA3 (84.1 ± 27.0) compared to BR   (71.6 ± 19.0)   - No difference in SWS latency, arousal index or stage shifts - Proportion of different NREM sleep stages in relation to TST did not change - MSLT: no significant difference in SL of individual naps |
| BaHammam et al. (2005) | NRS | 101 | Healthy  Age: fasting Saudis 32.6 ± 1.5 y, fasting non-Saudis: 29.3 ± 1.6 y, non-fasting non-Saudis: 31.5 ± 2.3 y  61 males, 40 females  BMI: Fasting Saudis 23.7 ± 1.0, fasting non-Saudis: 26.4 ± 1.1, non-fasting non-Saudis: 27.6 ± 2.1 kg/m^2^ | Beginning of fasting between 04:40 and 04:50, end of fasting between 17:05 and 17:20 during the month of Ramadan | ⬠ | Sleep survey   - sleep patterns - daytime sleepiness - meal habits - chronotyp   Epworth Sleepiness Scale | - No changes in TST or daytime sleepiness in all 3 groups - In fasting groups: bedtime and wake-up time were delayed significantly at RA1 and RA3 compared to BR - In non-fasting group, bedtime was delayed significantly during Ramadan - Sleep behavior changes during Ramadan in non-fasting |
| BaHammam et al. (2010) | prospective | 6 | Males, healthy  Age: 20.5 ± 2. 9 y  BMI: 22.6 ± 2.7 kg/m^2^ | Fasting at fixed times of the day during the month of Ramadan | ⬟ | Accelerometry   - TST - SE | - Wake up time was delayed during Ramadan compared to BR (12.68 ± 1.41 vs. 10.42 ± 1.87) - No change in TST - ↑ TST + nap time during Ramadan vs BR |
| Bahammam et al. (2013a) | prospective | 8 | Males, healthy  Age: 25.3 ± 2.9 y  BMI: 23.4 ± 3.2 kg/m^2^ | Fasting at fixed times of the day (18:45-04:00) during the month of Ramadan | ⬟ AND ⬠ | PSG   - TST - SE - SOL - WASO - Sleep staging   Epworth Sleepiness Scale | - ↓ REM sleep percentage (17.7 ± 8.1%) at Ramadan (18.6 ± 10.7%) compared to BR (25.6 ± 4.8%) (p < 0.05) - Bedtime and wake time were delayed during Ramadan (p < 0.05) - No significant differences in the TST, SOL and REM onset latencies - Arousal index and stage shifts did not differ significantly |
| BaHammam et al. (2013b) | NRS | 16 | Males, healthy  Age: fasters 36.2 ± 4.5 y; non‑fasters 34.8 ± 3.3 y  BMI: fasters 26.3 ± 2.4; non‑fasters 25.7 ± 3.4 kg/m^2^ | Fasting at fixed times of the day (04:05- 18:28) during the month of Ramadan | ⬟  AND  ⬠ | Accelerometry   - TST   Epworth Sleepiness Scale | - During Ramadan bedtime and wake time were delayed - ↓ TST for fasters (5.91 ± 1.36 h, 4.95 ± 1.46 h, and 4.78 ± 1.36 h during BR, RA1, and RA2, respectively, p < 0.001), but not for non‑fasters - No change in ESS score |
| Bahammam et al. (2014) | prospective | 8 | Males, healthy  Age: 26.6 ± 4.9 y  BMI: 23.7 ± 3.5 kg/m^2^ | During BLF: fasting during 7 days and no change in lifestyle and eating habits.  During the last 3 weeks, routine activities and eating habits.  Fasting at fixed times of the day during the month of Ramadan  After RA : no fast and no change in lifestyle and eating habits | ⬟ AND ⬠ | PSG   - TST - SE - SOL - WASO - Sleep staging   Epworth Sleepiness Scale  Multiple sleep latency test | - ESS score of 7.3 ± 2.7, no change the four study periods - Intermittent fasting results in ↓ REM sleep with no impact on other sleep stages, the arousal index or daytime sleepiness |
| BaHammam et al. (2016) | prospective | 8 | Males, healthy  Age: 26.6 ± 4.9 y  BMI: 23.7 ± 3.5 kg/m^2^ | Fasting at fixed times of the day (18:34- 04:00) during the month of Ramadan | ⬟ | PSG   - TST - SE - SOL - WASO - Sleep staging   Accelerometry   - TST | - Shift delay in bedtime and wake-up time during Ramadan - No change in TST, SOL, SE, arousals index |
| Bahijri et al. (2013) | prospective | 23 | Healthy  Age: 23.1 ± 1.2 y  18 males, 5 females  BMI: 24.6 ± 1.21 kg/m^2^ | Fasting at fixed times of the day during the month of Ramadan | ⬠ | Sleep survey | - ↓ TST - ↓ sleep quality |
| Bener et al. (2018) | prospective | 1246 | T2DM  Age: males 51.5 ± 12.6 y; females 49.3 ± 14.4 y  593 males, 653 females  BMI: ≤ 24.9 kg/m^2^ (n=326), 25–29.9 kg/m^2^ (n=574), ≥ 30–34.9 kg/m^2^ (n=346) | Fasting at fixed times of the day (02:45 -21:45) during the month of Ramadan | ⬠ | Sleep survey | - ↓ TST (during 5.61 ± 0.58 h vs. after Ramadan 6.93 ± 0.72 h; p < 0.001) |
| Bener et al. (2021) | prospective | 1118 | Hypertension  Age: males 51.5 ± 12.6 y; females 49.1 ± 14.4 y  593 males, 653 females  BMI : ≤ 24.9 kg/m^2^ (n=295), 25–29.9 kg/m^2^ (n=517), ≥ 30–34.9 kg/m^2^ (n=306) | Fasting at fixed times of the day during the month of Ramadan | ⬠ | Sleep survey | - Fasting during Ramadan has no effect on sleep quality and fatigue - Between BR and AR: ↓ TST in males -1.30 (-1.80; -0.80) p<0.001 and in female -1.22 (-1.77; -1.54) p<0.001 |
| Boujelbane et al. (2022) | NRS | 58 | Healthy  Age: 62.9 ± 4.0 y  27 males, 31 females  BMI active group: 26.6 ± 2.8; control group 27.3 ± 2.7 kg/m^2^ | Fasting at fixed times of the day during the month of Ramadan | ⬠ | PSQI  Epworth Sleepiness Scale | - In both group Global PSQI and ESS scores : poor sleep quality and excessive daytime sleepiness |
| Boukhris et al. (2019) | prospective | 13 | Males, physically active Age: 21.2 ± 2.9 y  BMI: < 25 kg/m^2^ | Fasting at fixed times of the day during the month of Ramadan | ⬠ | PSQI | - Periods effect on total score of PSQI (p < 0.05) - ↑ PSQI at RA2 in comparison with BR and RA4 - No effect on SOL and SE - ↑ TST duration at RA2 and RA3 in comparison with BR and AR (p< 0.05) |
| Boukhris et al. (2022) | prospective | 15 | Males, physically active Age: 21.0 ± 3.0 y  BMI: < 25 kg/m^2^ | Fasting at fixed times of the day during the month of Ramadan | ⬠ | PSQI | - ↓ TST - ↑ PSQI score |
| Bouzid et al. (2019) | prospective | 8 | Males, elite soccer players Age: 21.0 ± 0.4 y  BMI: < 25 kg/m^2^ | Fasting at fixed times of the day during the month of Ramadan | ⬠ | Sleep survey   - Sleep quality | - No difference between BR and RA4 in quality of sleep |
| Brini et al. (2021a) | RCT crossover | 24 | Males, professional basketball players  Age: intervention group: 25.3 ± 2.6 y; control group: 24.8 ± 1.6 y  BMI: 23.4 ± 1.2 kg/m^2^ | Fasting at fixed times of the day during the month of Ramadan | ⬠ | PSQI | - ↓ SOL, sleep duration, and total score of PSQI, and ↑ in SE at the end of Ramadan (p < 0.01) |
| Brini et al. (2021b) | prospective | 24 | Professional basketball players  Age: males 26.9 ± 3.0 y; females 24.8 ± 1.8 y  12 males, 12 females  BMI males: 23.2 ± 1.1; females 24.1 ± 1.5 kg/m^2^ | Fasting at fixed times of the day during the month of Ramadan | ⬠ | Sleep survey   - TST - Sleep quality | - ↓ sleep quality at RA4 - ↑ TST between RA1 and RA4 (p < 0.01) |
| Celik et al. (2022) | cross-sectional | 32 | Healthy  Age: 19-32 y  12 males, 20 females  BMI males 24.9 ± 3.0; females 24.1 ± 3.8 kg/m^2^ | Fasting at fixed times of the day during the month of Ramadan | ⬠ | PSQI | - No changes were observed in sleep quality |
| Chamari et al. (2012) | GRT | 42 | Males, professional soccer players  Age: 24 ± 4 y  BMI: < 25 kg/m^2^ | Fasting at fixed times of the day (04:00-19:15) during the month of Ramadan for two consecutive seasons (i.e. from 10 August to 11 September 2010 and from 1 to 30 August 2011) | ⬠ | Hooper’s index  Sleep | - No difference between fasting and NF players in quality of sleep - Sleep schedule was greatly modified with players not going to bed before 03.00 h |
| Chamari et al. (2016) | prospective | 11 | Males, healthy trained cyclists  Age: 21.6 ± 4.8 y  BMI: < 25 kg/m^2^ | Fasting at fixed times of the day during the month of Ramadan | ⬟ | PSG   - TST - SE - SOL - WASO   Sleep staging | - No change in TST - ↑ number of awakenings and light sleep duration during Ramadan (RA1 and RA4) - ↓ duration of deep and REM sleep stages after Ramadan |
| Chennaoui et al. (2009) | prospective | 8 | Males, middle-distance athletes  Age: 25.0 ± 1.3 y  BMI: ND | Fasting at fixed times of the day (05:00 -19:00) during the month of Ramadan | ⬠ | Sleep diary   - TST | - Nocturnal sleep time was lower at day 21 of RA than BR (p < 0.05) - At the end of RA (day 31): ↑ fatigue score on the Profile of Mood States questionnaire (p < 0.001) |
| Farooq et al. (2015) | prospective | 18 | Males, healthy  Age : 12.6 ± 1.5 y  BMI: ND | Maximum fasting duration of 14 hours during the month of Ramadan | ⬠ | Sleep survey  for 48h   - bed time - wake time - time taken to get to sleep   PSQI | - During week 4 of Ramadan: ↓ TST by 1.8 h - No change in PSQI scores during Ramadan month - No change in SE before Ramadan (85.9%), RA1 (88%), and RA4 (87.7%) - SE markedly ↑ 2 weeks after Ramadan (94.5%; P<0.001) |
| Graja et al. (2021) | prospective | 12 | Females, handball athletes Age: 16.5 ± 0.5 y  BMI: ND | Fasting at fixed times of the day (03:40 -19:23) during the month of Ramadan | ⬠ | Epworth Sleepiness Scale | - ↑ ESS scores during RA4 (13.3 ± 2.6) compared to BR (9.8 ± 2.6) (p < 0.05) - No significant difference between BR and RA1 |
| Habib et al. (2009) | cross-sectional | 243 | Patients on anti-retroviral therapy  Age: fasting: 35.5 ± 8.3 y; non-fasting 37.5 ± 8.8 y  117 males, 126 females  BMI: ND | Fasting at fixed times of the day during the month of Ramadan | ⬠ | Sleep survey | - ↓ TST - Delayed sleep schedules |
| Haouari-Oukerro et al. (2011) | prospective | 38 | Males, healthy  Age: 20.8 ± 1.0 y  BMI: 23.33 ± 0.28 kg/m^2^ | Fasting at fixed times of the day during the month of Ramadan | ⬠ | Sleep survey | - Bedtime was significantly delayed during Ramadan (p<0.01) - ↓ in TST (p<0.01) |
| Herrera et al. (2012) | prospective | 9 | Males, football players Age: 26 ± 4 y  BMI: ND | Fasting at fixed times of the day during the month of Ramadan | ⬠ | PSQI  Insomnia Severity Index  Epworth Sleepiness Scale | - During Ramadan:↓ TST (5.3±1.4 h) compared with BR (6.6±1.5 h, effect size=0.89) - Non-significant ↑ SOL (effect size=70.45) - Non-significant ↓ SE (effect size=0.69) |
| Hsouna et al. (2019) | prospective | 12 | Males, physically active Age: 21.9 ± 2.4 y  BMI: < 25 kg/m^2^ | Fasting at fixed times of the day during the month of Ramadan | ⬠ | PSQI | - Shorter TST and improved sleep quality following compared to during Ramadan - Sleep quality was lower at BR compared to RA1 (p<0.05), AR (p<0.01) and at RA4 and AR compared to RA1 (p<0.05) - ↑ TST at RA1 (p<0.05) and ↓ AR (p<0.05) compared to BR - Greater sleep disturbances at BR compared to RA1 (p<0.01), RA4 (p<0.01), and AR20 (p<0.05) and at AR compared to RA1 and RA4 (p<0.05) |
| Hsouna et al. (2020a) | prospective | 14 | Males, physically active Age: 22 ± 3 y  BMI: < 25 kg/m^2^ | Fasting at fixed times of the day during the month of Ramadan | ⬠ | PSQI | - No significant effect for SOL, SE or sleep disturbances - Sleep quality ↑ significantly at DR (p < 0.01) and AR (p < 0.05) in comparison with BR - TST ↓ DR in comparison with BR (p < 0.05) - PSQI score ↑ significantly |
| Hsouna et al. (2020b) | prospective | 12 | Males, physically active  Age: 21.1 ± 3.2 y  BMI: < 25 kg/m^2^ | Fasting at fixed times of the day during the month of Ramadan | ⬠ | PSQI | - Total PSQI scores ↑ during (p < 0.0005) and after (p < 0.0005) Ramadan in comparison to BR |
| Kadri et al. (2002) | prospective | 100 | Males, healthy  Age: 32 6 ± 5.8 y  BMI: ND | Fasting at fixed times of the day during the month of Ramadan | ⬠ | Hamilton Anxiety Scale   - TST   Habit of naps | - ↓ TST during the fasting month |
| Karli et al. (2007) | prospective | 10 | Males, elite power athletes  Age: 22.3 ± 1.3 y  BMI: 24.3 ± 1.7 kg/m^2^ | Fasting at fixed times of the day during the month of Ramadan | ⬠ | Sleep survey | - No significant differences in TST (p=0.15) |
| Khalfallah et al. (2004) | prospective | 148 | Males, healthy  Age > 18 y  BMI: ND | Fasting at fixed times of the day during the month of Ramadan | ⬠ | Sleep survey   - TST | - TST < 6 h in 66% of studied population - ↓ TST (p < 0.001) |
| Laraqui et al. (2012) | prospective | 2171 | Health care workers  Age: 41.8 ± 8.2 y  1126 males, 1044 females  BMI: ND | Fasting at fixed times of the day during the month of Ramadan | ⬠ | Sleep survey   - TST - Sleep quality   Epworth Sleepiness Scale | - During RA as compared to BR:   ↓ TST (6.8 ± 1.2 h vs 7.7 ± 1.4 h)  ↓ nocturnal sleep (6.1 ± 0.9 h vs 7.6 ± 1.3 h) ↑ naps (1.3 ± 0.9 h vs 0.5 ± 0.3 h)   - Bedtime and wake time were significantly delayed - ↑ Insomnia prevalence (48 versus 33%) - ↑ ESS > 10 prevalence (46 vs 18.1%), sleepiness at work (49.8 vs 19.4%), and sleepiness while driving (17.3 vs 9.8%) |
| Lee et al. (2019) | prospective | 16 | T2DM  Age: 58.5 ± 8.1 y  8 males, 8 females  BMI: ND | Fasting at fixed times of the day during the month of Ramadan | ⬟ | Accelerometry   - TST - Total time awake | - ↓ TST during Ramadan than BR period (mean 293 vs 349 min, respectively; p = 0.04) - Males reporting shorter TST than females (mean TST 228 vs 360 min; p = 0.031) |
| Leiper et al. (2008) | NRS | 87 | Males, football players  Age > 18 y  BMI: ND | Fasting at fixed times of the day during the month of Ramadan | ⬠ | Sleep survey   - TST - Sleep quality | - TST of non-fasting players ↓ 105 min sleep per night during RA1, before reverting back to their pre-Ramadan amount of sleep - TST of fasting players ↓ 60 min sleep per night throughout Ramadan - No change in sleep quality |
| Lessan et al. (2018) | prospective | 29 | Healthy  Age: 19–52 y  13 males, 16 females  BMI < 30 kg/m^2^ | Fasting at fixed times of the day during the month of Ramadan | ⬟ | Accelerometry   - TST | - During the Ramadan ↓ overall sleeping time and different sleeping patterns (waking up later in the morning, broken sleep to eat, not sleep at all during the night, afternoon nap) |
| Lipert et al. (2021) | prospective | 32 | Males, professional medium-distance runners Age: 28 ± 6.7 y  BMI: < 25 kg/m^2^ | Fasting at fixed times of the day (approximately 15 h) during the month of Ramadan | ⬠ | PSQI | - Sleep quality DR was poorer than BR (p < 0.05) - ↑ SOL DR (p < 0.001) and daytime disfunctions intensified (p < 0.05) - No differences in TST, habitual SE, sleep disturbances and use of sleeping medications |
| Magdy et al. (2020) | Cross- sectional | 430 | Epilepsy  Age: 31 y (IQR: 22-40)  219 males, 211 females  BMI: ND | Fasting at fixed times of the day during the month of Ramadan | ⬠ | PSQI | - Patients achieving successful Ramadan fasting had more efficient and longer sleep hours (8h [7-9] *vs* 7h [ 6-8], p < 0.001) |
| Margolis et al. (2004) | prospective | 137 | Healthy students  Age: 19 to 23 y  39 males, 98 females  BMI: ND | Fasting at fixed times of the day during the month of Ramadan | ⬠ | Sleep survey  Epworth Sleepiness Scale | - No variation in sleep score pre-Ramadan (10.04 ± 3.47), during Ramadan (10.46 ± 3.57), and post Ramadan (9.73 ± 3.33), p = 0.280 - Night sleep hours longer both before (6.22 ± 1.45) and after (6.22 ± 1.59) than during Ramadan (5.22 ± 1.85), p < 0.001 - Daytime sleep hours pre-Ramadan (1.05 ± 1.36) and post Ramadan (0.70 ± 1.21) shorter than during Ramadan (1.48 ± 1.46; p < 0.001) |
| Masood et al (2018) | Cross-sectional | 279 | Pregnant females who fasted during Ramadan Age: 25.9 ± 5.1 y  BMI: ND | Fasting at fixed times of the day during the month of Ramadan | ⬠ | Sleep survey | - 74% of participants ↓ TST to about 3-4 h |
| Meckel et al. (2008) | prospective | 19 | Males soccer players  Age: 15.1 ± 0.9 y  BMI: ND | Fasting at fixed times of the day during the month of Ramadan | ⬠ | Sleep survey   - TST | - No significant change in sleeping hours before (8.6 ± 0.7h) and during (8.6 ± 0.5h) Ramadan |
| Nassar et al. (2021) | GRT | 901 | T2DM and T1DM  Age: control group 51.3 ± 8.1 y; intervention group 47.8 ± 10.1 y  443 males, 458 females  BMI: ≤ 24.9 kg/m^2^ (n=46), 25–29.9 kg/m^2^ (n=723), ≥ 30–34.9 kg/m^2^ (n=132) | Fasting at fixed times of the day during the month of Ramadan | ⬠ | Sleep survey | - No change in TST |
| Nugraha et al. (2017) | NRS | 50 | Males, healthy  Age: 26.1 ± 1.0 y  BMI: 24.8 ± 0.7 kg/m^2^ | Fasting at fixed times of the day during the month of Ramadan | ⬠ | Epworth Sleepiness Scale | - ↑ ESS (p<0.01) from RA2 to RA4 in the fasting group |
| Roky et al. (2001) | prospective | 8 | Males, healthy  Age: 20-28 y  BMI: ND | Fasting at fixed times of the day during the month of Ramadan | ⬟ | PSG   - TST - SE - SOL - WASO   Sleep staging | - During Ramadan ↑ SOL, ↓ sleep period time and TST BR and RA4 - Proportion of NREM sleep ↑ during Ramadan and its structure changed, with an ↑ in stage 2 proportion and a ↓ in SWS duration - ↓ REM sleep duration and proportion during Ramadan |
| Roky et al. (2003) | prospective | 8 | Males, healthy  Age: 20-28 y  BMI: ND | During non-fasting days (BR, AR), meals were scheduled as follows: breakfast at 08:00 h, lunch at 12:30 h, light snack at 17:00 h and dinner at 20:30 h. During Ramadan, neither ate nor drank during the daytime. Meals schedule was: break of fasting at 18:00, light snack at 21:00 and dinner at 22:30 | ⬟ | PSG   - TST - SE - SOL - WASO - Sleep staging   Multiple sleep latency test | - ↓ TST (418 ± 12 versus 362 ± 26 min), ↑ SOL (27.7 ± 9 versus 85.1 ± 29 min) at the end of Ramadan - NREM sleep during Ramadan ↑ duration in stage 2 and ↓ in SWS - ↓ REM sleep duration and proportion during Ramadan |
| Romdhani et al. (2022) | cross-sectional | 1681 | Healthy athletes (41% elite, 51% team sport)  Age: 25.1 ± 8.7 y  1042 males, 639 females  BMI: ND | Fasting at fixed times of the day during the month of Ramadan during lockdawn | ⬠ | PSQI | - Longer (p < 0.001) and later (p < 0.001) daytime naps - ↓ sleep quality |
| Rosmelia et al. (2019) | prospective | 40 | Males, healthy  Age: 19.2 ± 1.0 y  BMI: 225 ± 4.0 kg/m^2^ | Fasting at fixed times of the day (04:25- 17:30) during the month of Ramadan | ⬠ | PSQI | - No reduction in TST - ↓ night sleep duration during Ramadan (p=0.000) associated with more subjects poor sleep quality (p=0.039) |
| Saddoud et al. (2021) | prospective | 14 | Males, Kung-Fu athletes Age: 19 ± 3 y  BMI: ND | Fasting at fixed times of the day during the month of Ramadan | ⬠ | PSQI  Epworth Sleepiness Scale  100 mm visual analogue scale   - Fatigue - Alertness   Concentration | - During Ramadan: higher sleep quality scores, lower TST and SE - No difference in SOL, sleep disturbance, daytime dysfunction, use of sleeping medications and total PSQI - ↑ ESS from 5.6 ± 2.9 at BR to 7.1 ± 3.5 at DR (21%, p = 0.004) - ↓ TST, sleep quality, attention, and concentration during RA |
| Tian et al. (2011) | prospective | 18 | Males, healthy  Age: 20.9 ± 3.3 y  BMI: ND | Fasting at fixed times of the day during the month of Ramadan | ⬠ | Sleep survey | - ↓ TST (p=0.005) - ↑ duration of daytime naps (p=0.001) |
| Trabelsi et al. (2021) | prospective | 68 | Males, amateur team sport referees  Age: 31.1 ± 10.8 y  BMI: ND | Fasting at fixed times of the day during the month of Ramadan | ⬠ | PSQI  Epworth Sleepiness Scale | - ↑ PSQI and ESS scores during Ramadan (p < 0.001) with 83.3% of participants scoring ≥ 5 in the PSQI - ESS score ≥ 16 in 3.8% before vs. 7.7% during Ramadan (p < 0.001) - ↓~ 1 h of TST during Ramadan (p < .001) associated with a delay in bedtime of ~ 2 h (p < 0.001) and of wake-up time of ~ 1 h (p < 0.001) - ↑ Daytime dysfunction and subjective sleep perception (p < 0.001) ↓ sleep medication (p = 0.041) |
| Waterhouse et al. (2009) | prospective | 20 | Males, healthy  Age: 18 y  BMI: < 25 kg/m^2^ | Fasting from 31 August to 30 September 2008, between sunrise at 05:30 and sunset at 19:30 | ⬠ | Sleep survey   - TST - Sleep quality | - Participants went to bed and rose later in Ramadan - Total time spent in bed ↓ from 10 h 05 min on control days to 7 h 59 min in Ramadan (p < 0.0005) |
| Wilson et al. (2009) | prospective | 14 | Males, professional soccer players  Age: 25 + 3.4 y  BMI: < 25 kg/m^2^ | Fasting at fixed times of the day during the month of Ramadan | ⬠ | Sleep survey | - ↑ sleep length (99 ± 42 min) during Ramadan - Timing of sleep was delayed significantly during Ramadan, mean delay in bedtime (199 ± 47 min) |
| Zerguini et al. (2007) | prospective | 55 | Males, professional soccer players  Age: 17–34 y  BMI: ND | Fasting at fixed times of the day during the month of Ramadan | ⬠ | Sleep survey | - No change of TST during Ramadan, ↓ quality of sleep |
| ***** Intermittent Fasting *** (n=16)** | | | | | | | |
| Bains et al. (2021) | Prospective | 16 | Healthy  Age: 34.0 ± 11.7 y  9 males, 6 females  BMI: 27.4 ± 1.3 kg/m² | Four-week fasting protocol (16:9 time-restricted feeding) | ⬠ | PSQI | - No significant changes in sleep quality |
| Cienfuegos et al. (2022) | RCT | 49 | BMI > 30 kg/m²  Age:  G1: 4h-TRF (49 ± 2 y) G2: 6h-TRF (46 ± 3 y) G3: control (45 ± 2y)  Body weight:  G1: 4h-TRF (101.0 ± 4.8 kg)  G2: 6h-TRF (99.3 ± 4.6 kg)  G3: control (92.7 ± 4.5 kg)  3 males, 46 females | Two eating conditions for ten weeks : 4h-TRF (eat ad libitum from 3 to 7 pm daily, and fast from 7 to 3 pm (20-h fast) and 6h-TRF (eat ad libitum from 1 to 7 pm daily, and fast from 7 to 1 pm (18-h fast) and one control group (no diet advice) | ⬠ | PSQI  Insomnia Severity Index  Berlin questionnaire | - Outcomes were not changed significantly (no group × time interaction) |
| Gabel et al. (2019) | Prospective study (single arm) | 23 | Age: 50 ± 2 y  Bodyweight: 95 ± 3.0 kg  3 males, 20 females  BMI > 30 kg/m² | 2 weeks baseline (diet and exercise routine) and 12 weeks TRF intervention (ad libitum from 10:00 am to 18:00 pm daily, and fasting from 18:00 pm to 10:00 am daily) | ⬠ | PSQI  Insomnia Severity Index  Berlin questionnaire | - PSQI total score was below 5 at week 1 (4.7 ± 0.5) and week 12 (4.8 ± 0.7) = good sleep quality - Unchanged in insomnia, sleep apnea and subjective measures of wake time, bedtime and TST |
| Hutchison et al. (2019) | Cross-over RCT | 15 | Males, healthy  Age: 55 ± 3 y  BMI: 33.9 ± 0.8 kg/m^2^ | One-week baseline, then randomized to two TRF protocols (7 days) crossover. TRFe (eating 8am-5pm) and TRFd (eating 12pm-9pm) | ⬟ | Accelerometry   - TST | - No effect on TST assessed by accelerometry |
| Kalam et al. (2021) | Prospective study (6 months) | 31 | BMI > 30 kg/m²  Age: 48 ± 2 y  6 males, 26 females | One-month baseline then six-month intermittent fasting (Alternate Day Fasting Low Carbs (ADF-LC): feast day then fast day) | ⬠ | PSQI  Insomnia Severity Index  Berlin questionnaire | - ADF-LC diet does not impact sleep quality, TST, insomnia severity or the risk of OSA |
| Kesztyus et al. (2020) | Prospective | 99 | Two groups: healthy students and BMI > 25 kg/m²  Age: 48.9 ± 1.1 y  16 males, 83 females  BMI 28.0 ± 5.7 kg/m^2^ | Three-month intervention : TRE (limit their daily food intake to 8–9 h and subsequently extend their nightly fasting period to 15–16 h) | ⬠ | Sleep survey   - TST - Sleep quality | - No change in TST - Sleep quality changed before and after TRE intervention |
| Kim et al. (2020) | Prospective study (4 weeks) | 15 | Healthy  Age: 36.8 ± 8.44 y  9 males, 6 females  BMI: 29.3 ± 4.6 kg/m² | Four-weeks intervention : TRE (2 meals per day, from 12pm to 8pm : 8h) | ⬟  AND  ⬠ | Korean PSQI  Insomnia Severity Index Epworth Sleepiness Scale Stanford Sleepiness Scale PSG   - TST - SE - SOL - WASO - Sleep staging | - No significant changes in results from questionnaires and scales - No changes in sleep structures in PSG before or after the program |
| Lopes et al. (2019) | Cross-sectional | 296 | Mild to severe OSA  Age: 20 to 60 y  211 males, 85 females  BMI: >25 kg/m^2^ | N/A | ⬟ AND ⬠ | PSG   - TST - SE - SOL - WASO - Sleep staging   PSQI  Epworth Sleepiness Scale | - Eating window was significantly associated with TST - People with an eating duration ≤ 12 hours presented higher self-reported TST than in individuals with eating duration > 12 hours |
| Lowe et al. (2020) | RCT | 116 | Age: 46.5 ± 10.5 y  70 males, 46 females  BMI: 32.7 ± 4.2 kg/m² | 12 weeks, two interventions (one group: recommendations on timing and TRE group: eat ad libitum 12:00 pm until 8:00 pm and fast from 8:00 pm until 12:00 pm the following day (16 hours fast:8 hours eat) | ⬟  AND  ⬠ | Sleep survey  Accelerometry   - SE - Daily movement - SOL - Energy expenditure | - No significant changes in any of the self-reported sleep measures in either group or between groups - Accelerometry data revealed significant improvement in SE, significant decrease in SOL score and in the TRE group and between groups |
| Michalsen et al. (2003) | Prospective | 13 | Healthy  Age: 41.2 ± 13.4 y  1 male, 12 females  BMI: 23.9 ± 4.2 kg/m² | Two weeks (13 days) : three pre-fasting day and seven fasting (<300 kcal/day) days and three days of reintroduction of food intake | ⬟  AND  ⬠ | Visual analogue scale   - Quality of sleep - TST - SOL - WASO   PSG   - TST - SE - SOL - WASO - Sleep staging   Periodic leg movement | - No significant changes in PSG except for periodic leg movements that decreased from a mean of 54.8 to 16.9 - ↑ TST (p<0.002), ↑ sleep quality (p<0.03) assessed by self-reported methods |
| Park et al. (2021) | Prospective | 33 | Healthy  Age: 22.5 ± 2.8 y  8 males, 25 females  BMI: 22.7 ± 2.7 kg/m² | Four weeks, 8h TRE each day | ⬠ | Sleep survey   - TST - Sleep quality | - Late-shifted sleeping patterns - No significant differences in TST and sleep quality |
| Parr et al. (2020) | Prospective | 19 | T2DM  Age: 50 ± 9 y  9 males, 10 females  BMI: 34.0 ± 5.0 kg/m² | Two-weeks baseline recording, then four-weeks TRE intervention (fast from 10h to 19h as many days as possible) | ⬠ | PSQI | - No changes were observed in sleep quality variables |
| Teong et al. (2021) | GRT | 46 | Females, healthy  Mean age : 50 y  BMI: 32.9 ± 4.4 kg/m² | Eight-weeks, 2 groups : CR, calorie restriction diet prescribed at 70% of calculated energy requirements; IF, intermittent fasting diet prescribed at 70% calculated energy requirements | ⬠ | PSQI | - No within-group and between-group differences in sleep quality |
| Wilkinson et al. (2020) | Prospective | 19 | Metabolic syndrome  Age: 59 ± 11.1 y  13 males, 6 females  BMI: 33.1 ± 4.7 kg/m² | 2-week baseline and 12-week intervention periods (TRE) | ⬟  AND  ⬠ | PSQI  Accelerometry   - TST   Sleep survey | - No significant changes - Modest changes in sleep from actigraphy coupled with significant changes in subjective rating of sleep immediately after waking up indicates TRE impacts sleep |
| Simon et al. (2022) | RCT | 20 | Age: TRE: 46.4 ± 12.4 y non-TRE: 44.2 ± 12.3 y  4 males, 16 females  BMI ≥ 25 kg/m^2^ | 12-week intervention  TRE (n = 11): 8-h eating window,  non-TRE (n = 9): typical eating habits | ⬟ | Accelerometry | - No significant changes in sleep schedules or TST |
| Xie et al. (2022) | RCT | 90 | Healthy  Age: eTRF: 28.7 ± 9.7 y mTRF: 31.1 ± 8.41 y  Control: 33.6 ± 11.6 y  26 males, 64 females  BMI: eTRF: 22.7 ± 3.1 kg/m^2^, mTRF: 21.4 ± 2.2 kg/m^2^, Control: 21.5 ± 2.9 kg/m^2^ | Three groups (eTRF – early, mTRF – midday, and control) | ⬠ | PSQI | - No significant differences in sleep quality among the three groups |
|  |  |  | ***** Day-to-day irregular meal pattern *** (n=3)** | | | |  |
| Beigrezaei et al. (2022) | Cross-sectional | 988 | Females, healthy  Age: 12-18 y  BMI: ≤ 24.9 kg/m^2^ (n=389), 25–29.9 kg/m^2^ (n=275), ≥ 30–34.9 kg/m^2^ (n=299) | N/A | ⬠ | Insomnia Severity Index | - Regular meal consumption was significantly associated to lower odds of insomnia |
| Faris et al. (2021) | Cross-sectional | 498 | Healthy students  Age: 18-30 y  185 males, 313 females  BMI: ≤ 24.9 kg/m^2^ (n=310), 25–29.9 kg/m^2^ (n=106), ≥ 30–34.9 kg/m^2^ (n=82) | N/A | ⬠ | PSQI | - Irregular mealtimes (r = − 0.094, p = 0.018) were correlated with poor sleep quality |
| Tahara et al. (2021) | Cross-sectional | 4490 | Healthy workers  Age: 47.4 ± 0.1 y  3291 males, 1199 females  BMI: 22.8 ± 0.1 kg/m² | N/A | ⬠ | PSQI (Japanese version) | - Sleep quantity and quality were negatively correlated with irregular meal timing - Scores of all factors in subjects’ sleep problems were higher in the participants with irregular meal timing |

ADF-LC: alternate day fasting low carbohydrate diet; AR: after Ramadan; BLF: baseline fasting; BMI: body mass index; BR: before Ramadan; BRUMS: Brunel Mood State; CI: confidence interval; CR: calorie restriction; DR: during Ramadan; ESS: Epworth Sleepiness Scale; eTRF: early time-restricted eating; FNS: fasting non Saudis; FS: fasting Saudis; GRT: group randomized trials; IF: intermittent fasting; IQR: interquartile range; KSS: Karolinska Sleepiness Scale; MSLT: multiple sleep latency test; mTRF: midday time-restricted eating; N/A: not applicable; ND: not done; NREM: non rapid-eye movement sleep; NRS: non randomized studies; OR: odd-ratio; PSG: polysomnography; PSQI: Pittsburgh Sleep Quality Index; RA: Ramadan; RA1: first week of Ramadan; RA2: second week of Ramadan; RA3: third week of Ramadan; RA4: fourth week of Ramadan; RCT: randomized controlled trials; REM: rapid-eye movement sleep; SE: sleep efficiency; SOL: sleep onset latency; SWS: slow wave sleep; T1DM: type 1 diabetes mellitus; T2DM: type 2 diabetes mellitus; TRE: time-restricted eating; TST: total sleep time; WASO: wake after sleep onset; ↓: decrease; ↑: increase; ⬟: objective measures; ⬠: self - reported measures

**Table S4.** Characteristics and main findings of studies exploring the effect of meal frequency on sleep

| Study | Design | Sample size | Participants’ characteristics | Intervention / duration | Sleep measurement and outcomes | | Main findings |
| --- | --- | --- | --- | --- | --- | --- | --- |
| Beigrezaei et al. (2022) | Cross-sectional | 988 | Females, healthy  Age: 12-18 y  BMI: ND | N/A | ⬠ | Insomnia Severity Index | - Frequency of meal intake was not associated with insomnia |
| Fuji et al. (2009) | Cross-sectional | 360 | Medical students  Age: 22.1 ± 2.2 y  215 males, 145 females  BMI: ND | One-shot questionnaires | ⬠ | PSQI  ESS | - Wake time was significantly later in both men and women eating less than 2 meals, compared to those eating more than 3 meals - In men, bedtime was significantly later for those eating 2 meals or less - In women, better sleep quality was found among those who ate meals 3 times daily |
| Reid et al. (2014) | Prospective | 59 | Healthy  Age: 31.7 ± 11.8 y  29 males, 30 females  BMI: 24.1 ± 4.2 kg/m² | Seven days assessment | ⬟ AND ⬠ | Accelerometry   - TST   Sleep survey | - Eating frequency was not correlated with TST |
| BMI: body mass index; ESS: Epworth Sleepiness Scale; N/A: not applicable; PSQI: Pittsburgh Sleep Quality Index; TST: total sleep time; ⬟: objective measures; ⬠: self - reported measures | | | | | | | |


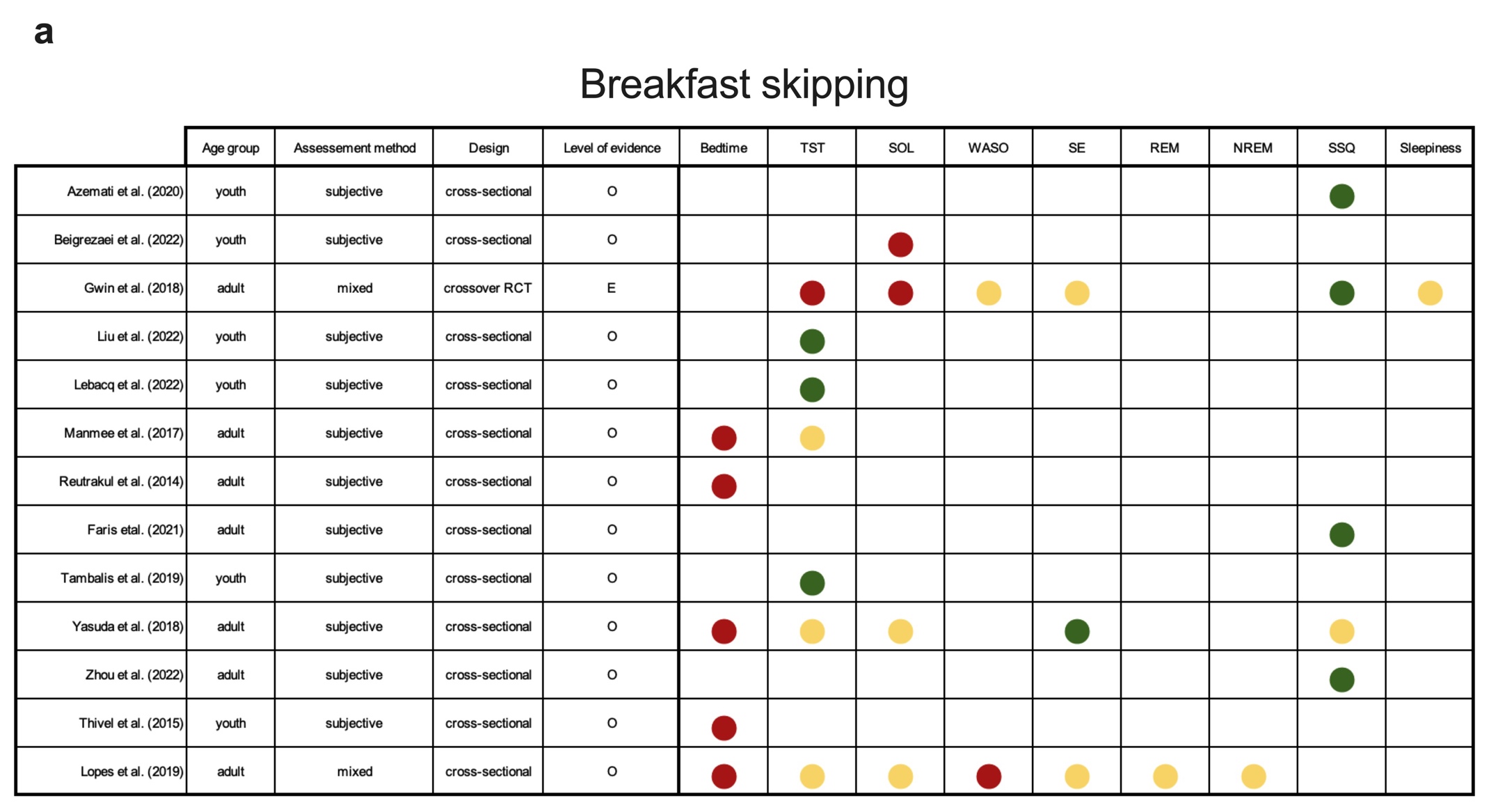


**Figure S1.** Meal timing dimension detailed tabular representation of each study sleep outcomes (continued)


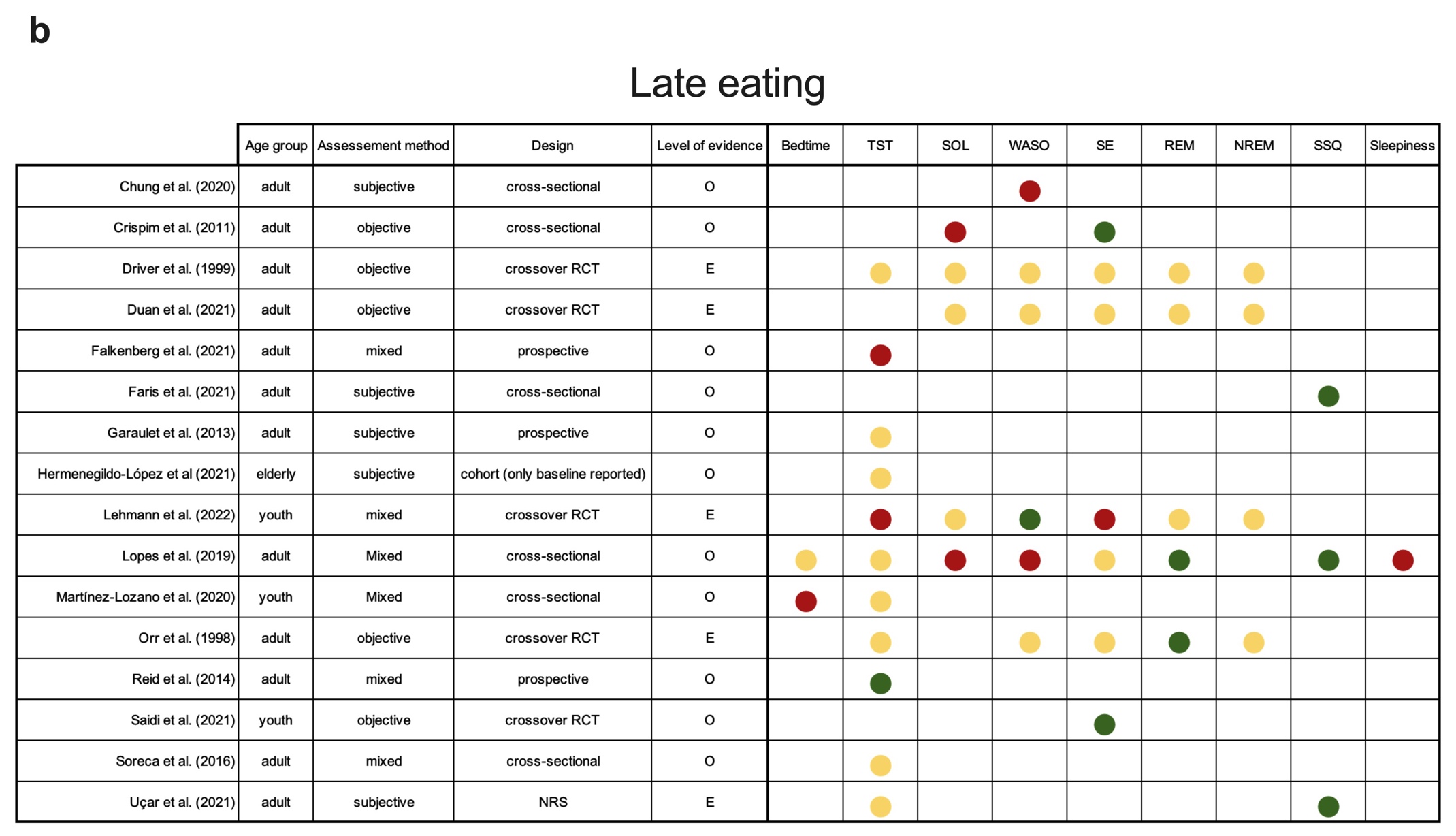


**Figure S1.** Meal timing dimension detailed tabular representation of each study sleep outcomes (continued)


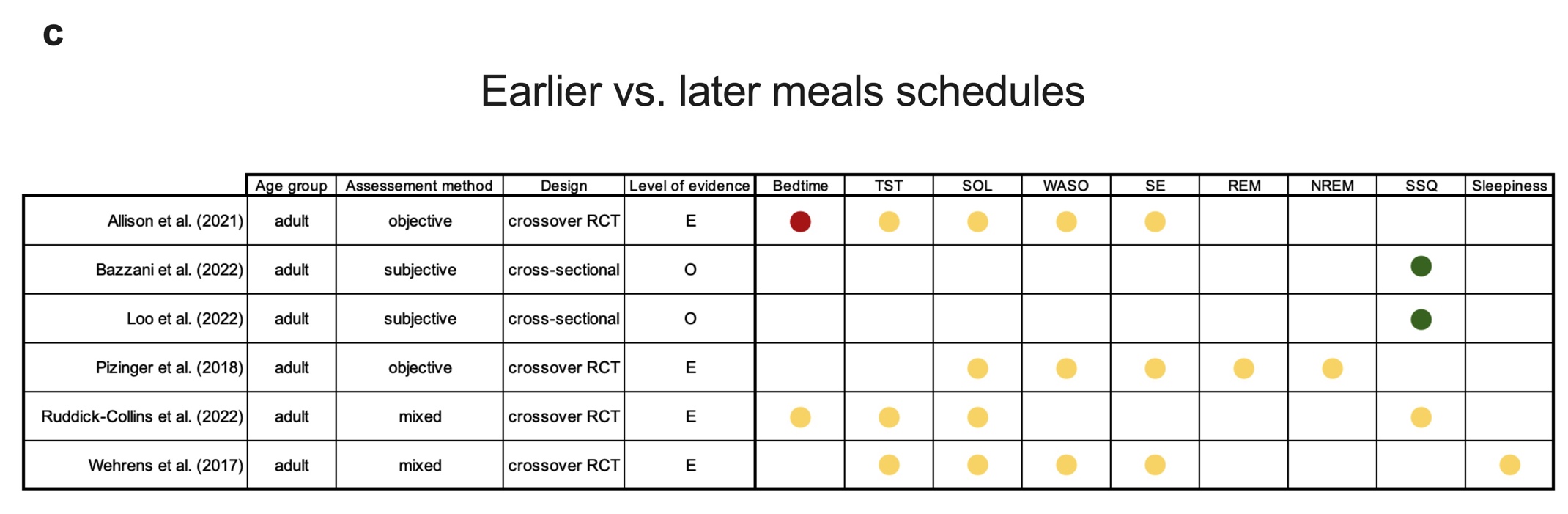


**Figure S1.** Meal timing dimension detailed tabular representation of each study sleep outcomes. E: experimental; NREM: non rapid-eye movement sleep; NRS: non randomized studies; O: objective; RCT: randomized controlled trial; REM: rapid-eye movement sleep; SE: sleep efficiency; SOL: sleep onset latency; SSQ: subjective sleep quality; SWS: slow wave sleep; TST: total sleep time; WASO: wake after sleep onset. Yellow color indicates no effect for each sleep variable, while green color indicates an increase and red a decrease. For bedtime, green color indicates an advance where red color indicates a delay.


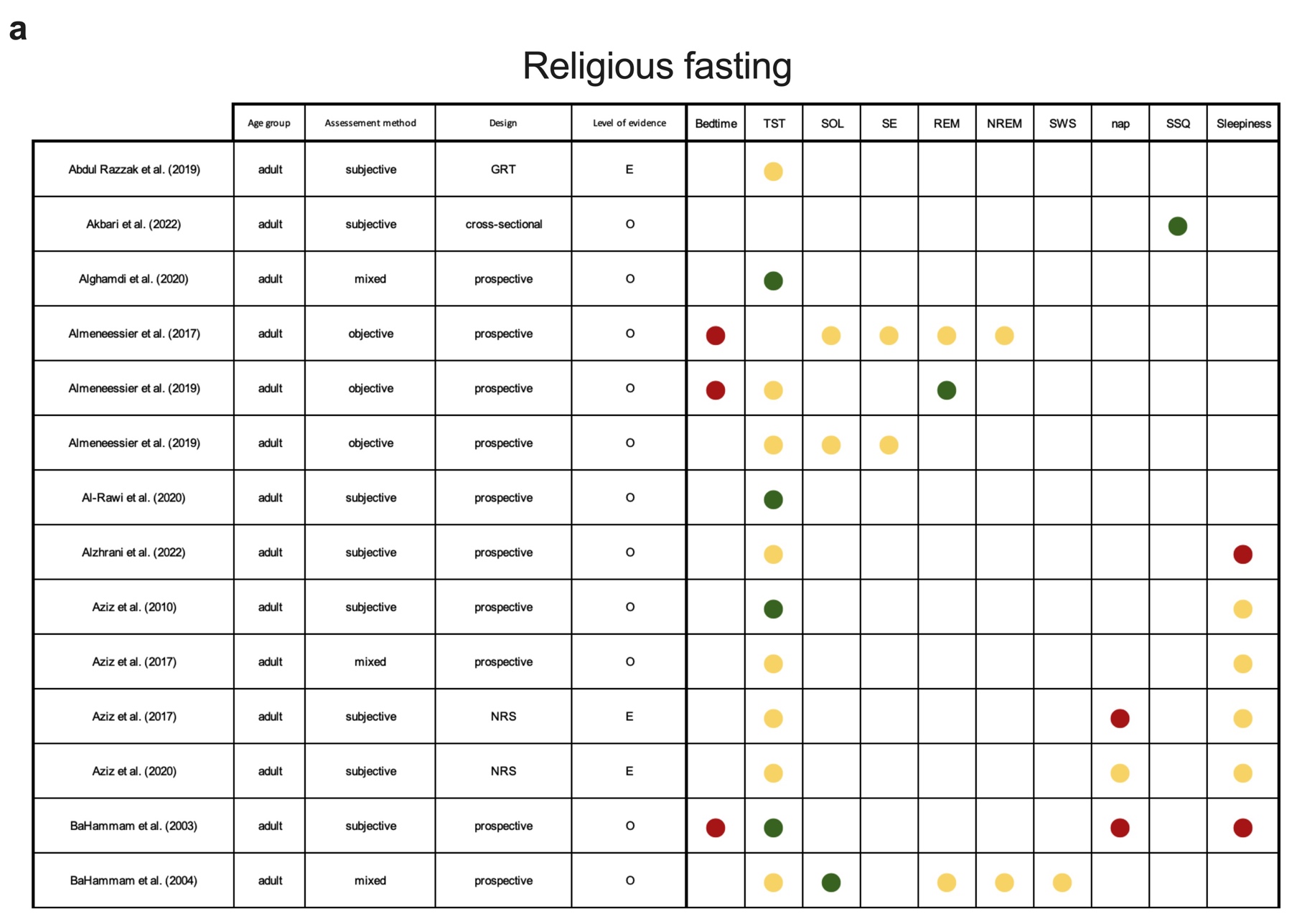


Diurnal fasting

**Figure S2.** Diurnal fasting and intermittent fasting sub-dimensions detailed tabular representation of each study sleep outcomes (continued)


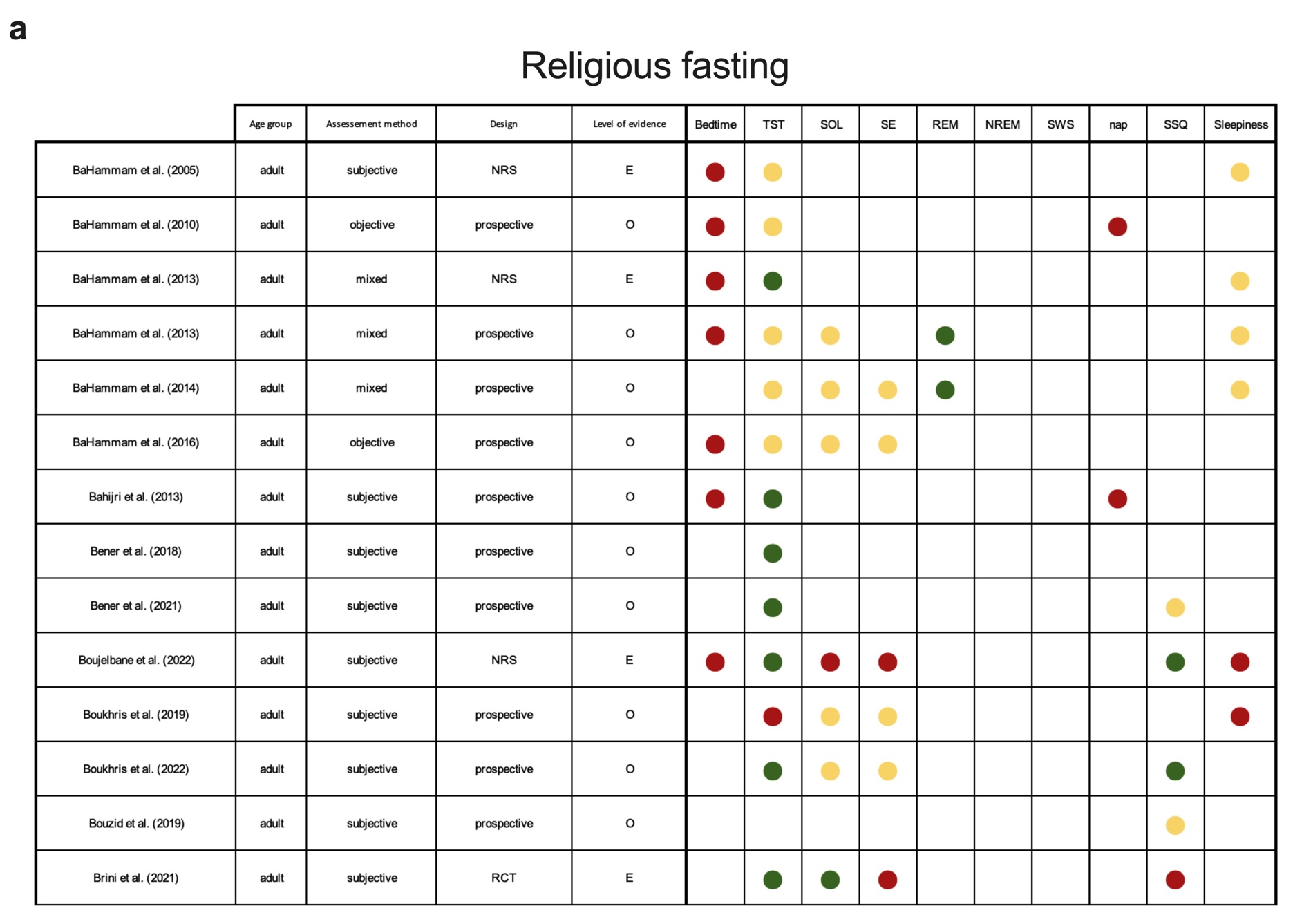


**Figure S2.** Diurnal fasting and intermittent fasting sub-dimensions detailed tabular representation of each study sleep outcomes (continued)


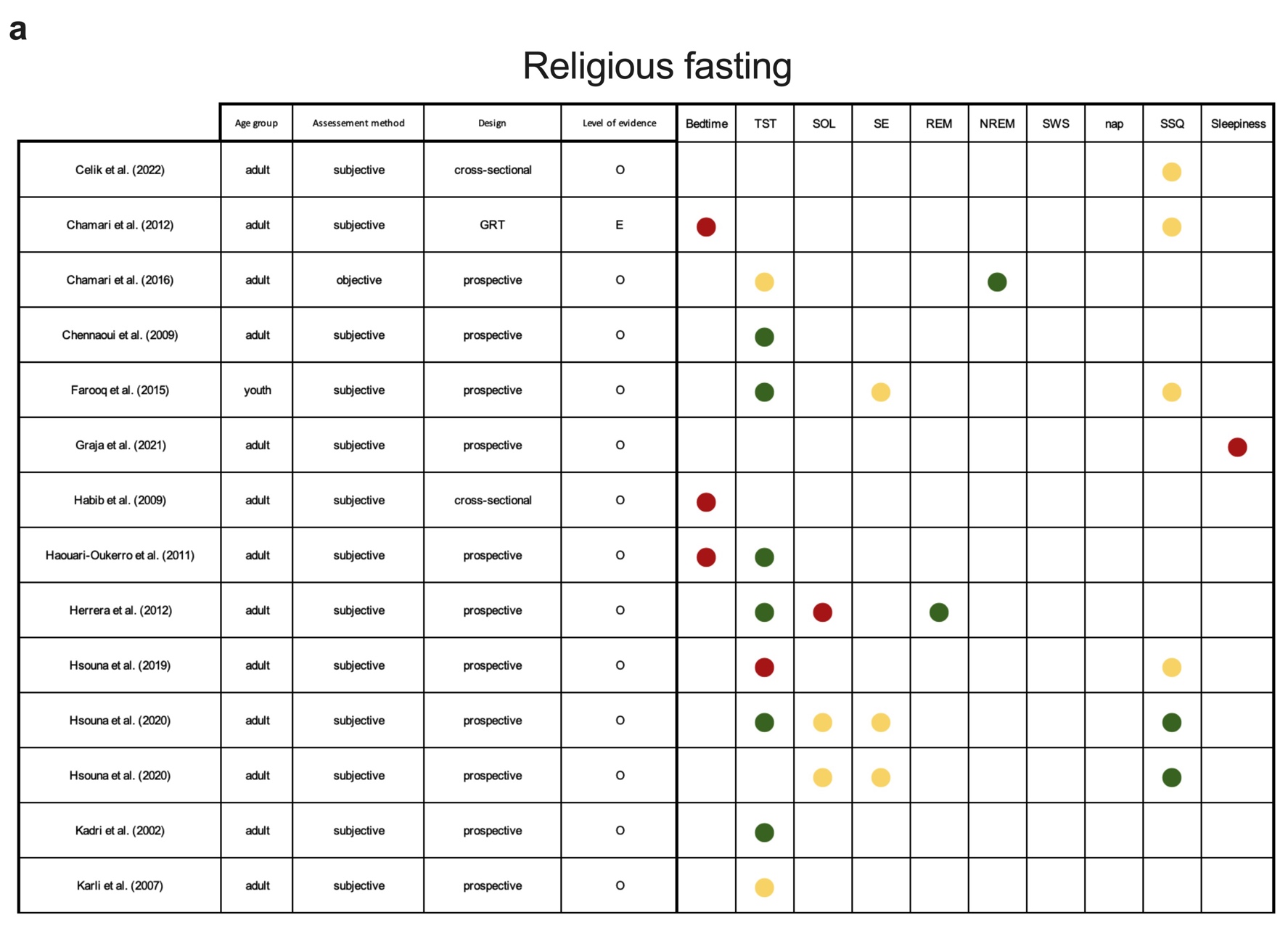


**Figure S2.** Diurnal fasting and intermittent fasting sub-dimensions detailed tabular representation of each study sleep outcomes (continued)


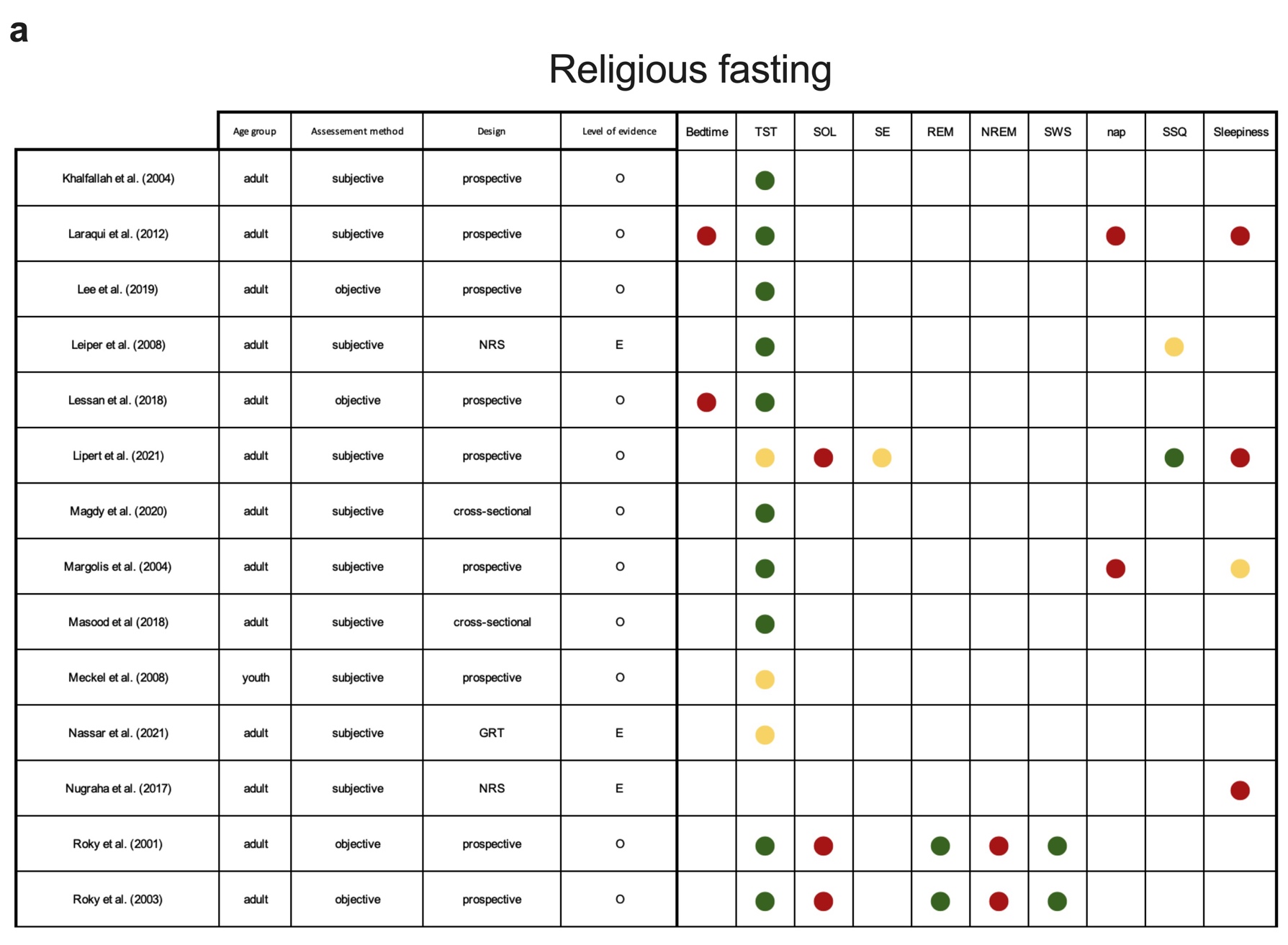


**Figure S2.** Diurnal fasting and intermittent fasting sub-dimensions detailed tabular representation of each study sleep outcomes (continued)


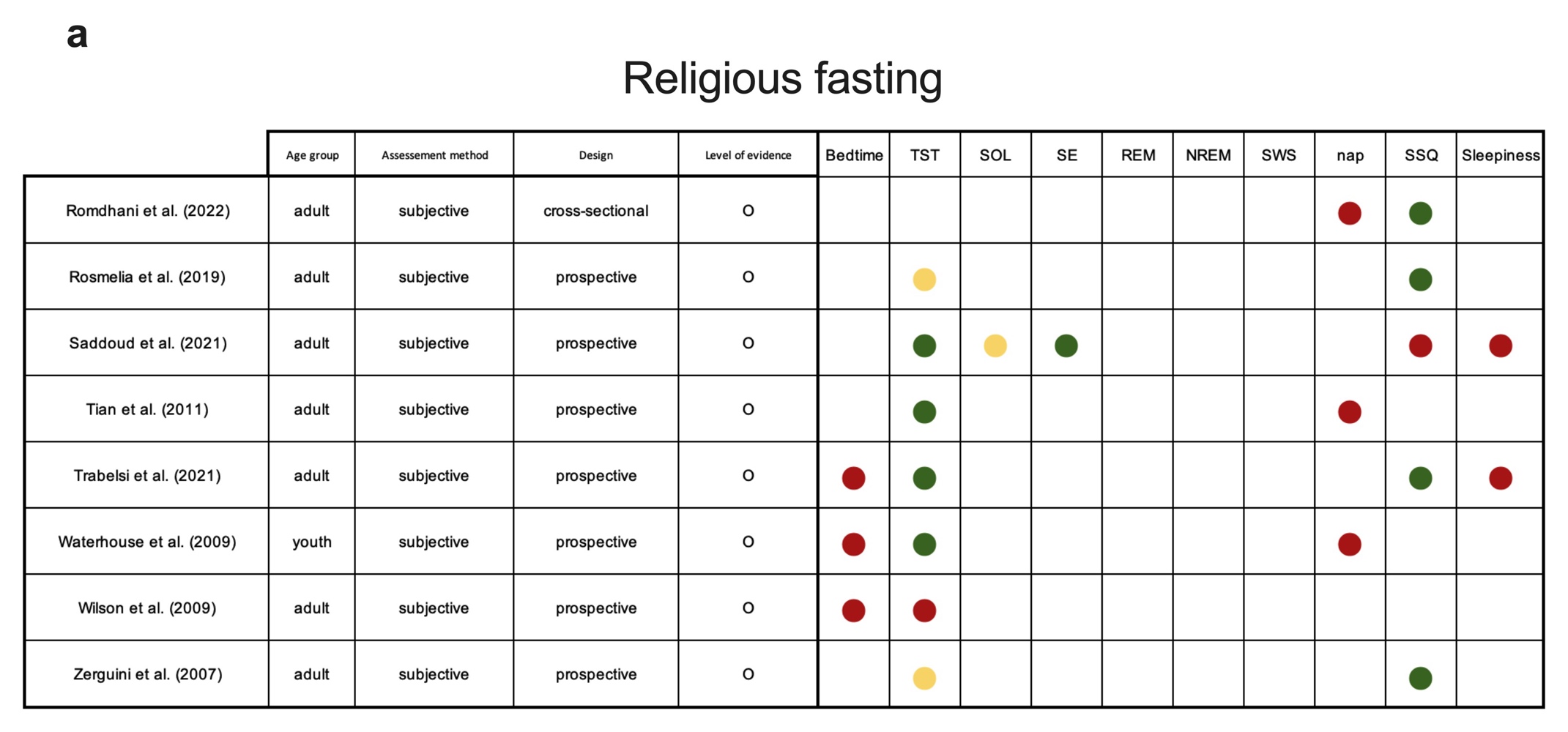


**Figure S2.** Diurnal fasting and intermittent fasting sub-dimensions detailed tabular representation of each study sleep outcomes (continued)


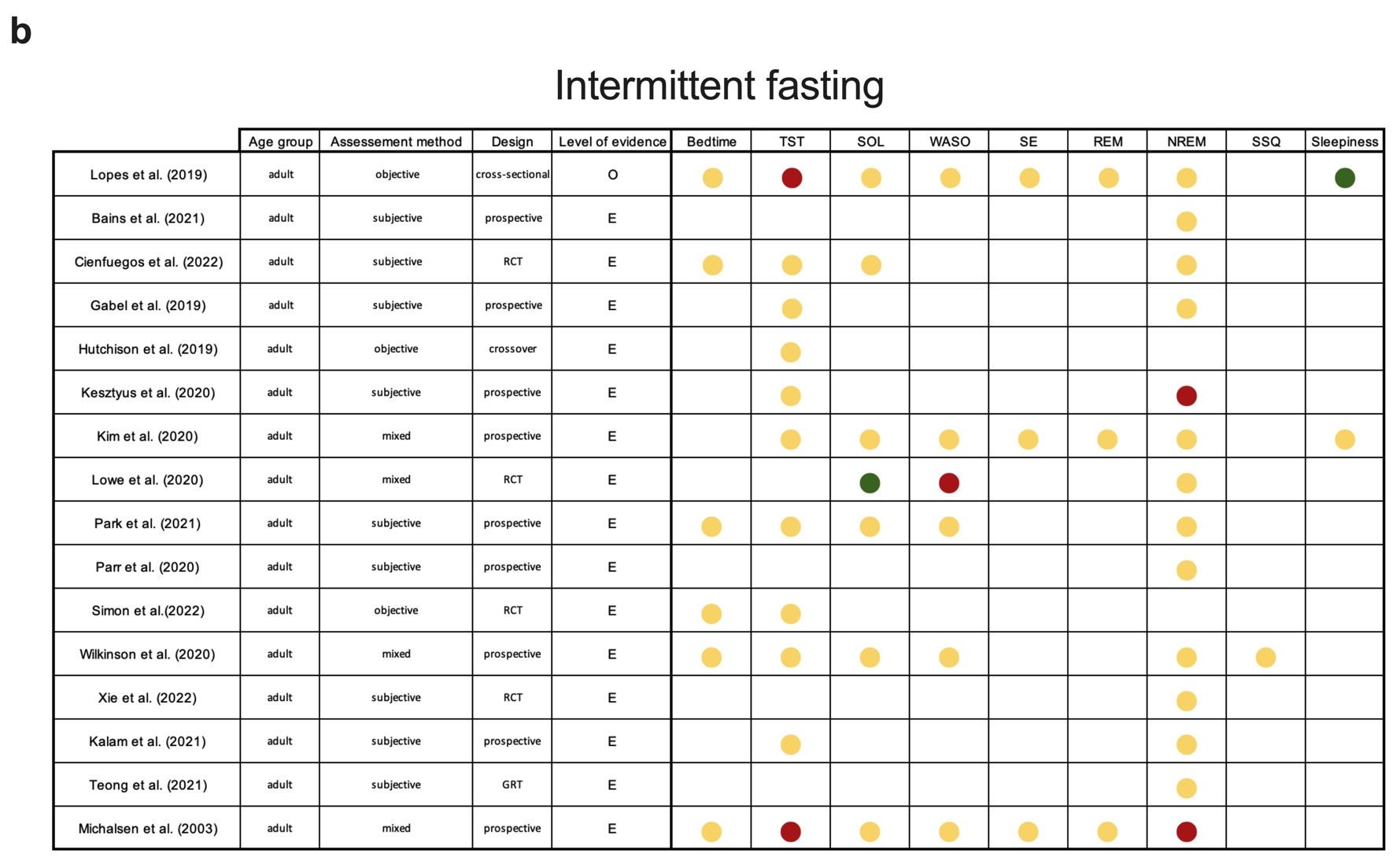


**Figure S2.** Diurnal fasting and intermittent fasting sub-dimensions detailed tabular representation of each study sleep outcomes. E: experimental; GRT: group randomized trials; NREM: non rapid-eye movement sleep; NRS: non-randomized studies; O: objective; RCT: randomized controlled trial; REM: rapid-eye movement sleep; SE: sleep efficiency; SOL: sleep onset latency; SSQ: subjective sleep quality; SWS: slow wave sleep; TST: total sleep time; WASO: wake after sleep onset. Yellow color indicates no effect for each sleep variable, while green color indicates an increase and red a decrease. For bedtime, green color indicates an advance, red color indicates a delay.
